# Identification of human peripheral blood monocyte gene markers for early screening of solid tumors

**DOI:** 10.1371/journal.pone.0230905

**Published:** 2020-03-30

**Authors:** Siyang Chen, Menghan Liu, Bowen Liang, Shanghua Ge, Jie Peng, Haiyue Huang, Yanmei Xu, Xiaoli Tang, Libin Deng

**Affiliations:** 1 Jiangxi Provincial Key Laboratory of Preventive Medicine, School of Public Health, Nanchang University, Nanchang, Jiangxi, P.R. China; 2 The Second Clinical Medical College of Nanchang University, Nanchang, Jiangxi, P.R. China; 3 School of Public Health (Shenzhen), Sun Yat-Sen University, Shenzhen, Guangdong, P.R.China; 4 School of Basic Medical Science, Nanchang University, Nanchang, Jiangxi, P.R. China; 5 The Second Affiliated Hospital of Nanchang University, Nanchang, Jiangxi, P.R. China; 6 Institute of Translational Medicine of Nanchang University, Nanchang, Jiangxi, P.R. China; Chang Gung Memorial Hospital at Linkou, TAIWAN

## Abstract

As cancer mortality is high in most regions of the world, early screening of cancer has become increasingly important. Minimally invasive screening programs that use peripheral blood mononuclear cells (PBMCs) are a new and reliable strategy that can achieve early detection of tumors by identifying marker genes. From 797 datasets, four (GSE12771, GSE24536, GSE27562, and GSE42834) including 428 samples, 236 solid tumor cases, and 192 healthy controls were chosen according to the inclusion criteria. A total of 285 genes from among 440 reported genes were selected by meta-analysis. Among them, 4 of the top significantly differentially expressed genes (*ANXA1*, *IFI44*, *IFI44L*, and *OAS1*) were identified as marker genes of PBMCs. Pathway enrichment analysis identified, two significant pathways, the ‘primary immunodeficiency’ pathway and the ‘cytokine-cytokine receptor interaction’ pathway. Protein- protein interaction (PPI) network analysis revealed the top 27 hubs with a degree centrality greater than 23 to be hub genes. We also identified 3 modules in Molecular Complex Detection (MCODE) analysis: Cluster 1 (related to *ANXA1*), Cluster 2 (related to *IFI44* and *IFI44L*) and Cluster 3 (related to *OAS1*). Among the 4 marker genes, *IFI44*, *IFI44L*, and *OAS1* are potential diagnostic biomarkers, even though their results were not as remarkable as those for *ANXA1* in our study. *ANXA1* is involved in the immunosuppressive mechanism in tumor-bearing hosts and may be used in a new strategy involving the use of the host's own immunity to achieve tumor suppression.

## Introduction

Numerous studies to date have emphasized the importance of early detection in cancer, such that treatment can be initiated as early as possible. Indeed, early detection of cancer is key to successful treatment and patient survival[1, 2].

Early screening is generally performed via testing in individuals with a high risk or high probability of tumor detection in early stages (secondary prevention) or to prevent complications (third-level prevention). The Cancer Screening Program aims to reduce the morbidity and mortality of cancer through early detection of malignant or precancerous lesions. However, the basic ethical dilemma of screening programs is that many people must be exposed to the burden and risk of intervention with little benefits [3]. In fact, the majority of existing screening approaches are invasive. For example, the highest recommended items listed in the US Preventive Services Task Force (USPSTF) Cancer Screening Guidelines (colon cancer and cervical cancer screening) are invasive techniques [4]. Invasive tools are not a satisfactory choice for weak patients, and healthy people with no symptoms are reluctant to undergo these procedures. In addition, most screening programs cannot recognize and diagnose tumors until they develop to a specific extent. As an example, if breast cancer is detected in the breast by palpation or mammography, it may have been present for several years, with the ability to spread to distant organs [5]. Accordingly, there is an urgent need to establish reliable tools for the identification of cancer at early stages, especially prior to the development of clinical symptoms.

Biomarkers have often been deemed an indicator of early tumor screening, and the effectiveness of their intervention in clinical diagnosis and monitoring has been confirmed many times. Early detection of cancer using effective biomarkers can facilitate more effective treatments, allowing patients to have better prognosis[6]. Tumor biomarkers include changes in tumor gene expression-specific mutations or promoter methylation that result in altered protein expression. Biomarkers produced by the tumor itself may be present in the adjacent body fluid or patient's blood circulation system, and this situation leads to a new strategy for establishing a minimally invasive early screening protocol for tumor detection. Furthermore, improvements in genomics and monitoring technologies have provided significant opportunities for cancer screening that make imaging more precise and more specific with regard to describing tumor biomarkers in the blood [7].

Recently, studies have shown that peripheral blood can carry information related to the presence of diseases, including prognosis and treatment response. Compared with existing approaches, cancer detection based on peripheral blood is more advantageous because of the easy accessibility and less invasive procedure for obtaining samples [8]. More importantly, tests based on blood diagnoses can result in better patient compliance for some cancers, such as colon cancer [9].

Peripheral blood mononuclear cells (PBMCs) are composed of immune cells, such as monocytes and lymphocytes. PBMCs are important players in the host immune defense system and can respond to various abnormalities in the host [10]. The development and survival of tumors is a complex process involving interaction between cancer cells, normal stromal cells, and host immune defense systems. The immune evasion mechanism of the tumor itself also has an important role. The main mechanism of tumor immune evasion is immunosuppression in the tumor microenvironment mediated by CD4+, CD25+, and FoxP3+ cells, regulatory T cells (Tregs) and other types of inhibitory cells [11]. Therefore, gene expression profiling of peripheral blood cells has potential in early cancer detection. The experimental results of Michael E. Burczynski et al. indicated that circulating monocytes of peripheral blood can be used as a surrogate monitor for tissues that are difficult to biopsy or a sensitive monitor to check the physiological state of the organism because they can migrate through various tissues of the body [12]. Additionally, Natalie C. Twine identified a group of PBMCs predictive genes that can distinguish between renal cell carcinoma (RCC) patients and normal volunteers with high precision. Furthermore, ongoing research by this group demonstrates that PBMCs from RCC patients can be accurately distinguished from the PBMCs of normal volunteers and also from those of patients with other types of solid tumors [13]. According to the characteristics of these cells, Praveen Sharma et al. showed that PBMCs can be used to develop gene expression-based tests for early detection of breast cancer [14]. The study by Michael K. Showe also suggests that the use of peripheral blood gene expression features to identify early non-small cell lung cancer (NSCLC) in high-risk populations is feasible and may reduce the number of patients who need to undergo biopsy or surgery to determine whether they have benign pulmonary nodules [15].

The rationale for using the PBMC transcriptome gene as a monitor for malignant solid tumors is based on the mechanism by which malignant growth causes characteristic changes in the blood biochemical environment. These changes are mostly related to the immune evasion mechanism of the tumor itself and will affect the expression pattern of some genes in blood cells. PBMC transcriptome gene expression is easily extracted as a tumor screening marker. Given their accessibility, PBMCs may provide potential predictive biomarkers in clinical pharmacogenomics [16].

In this study, we investigated solid tumors and selected human PBMC genetic alterations as a new screening program. We believe that blood-based surrogate markers may serve as accessible biomarkers for early detection, diagnosis, prognosis and prediction of cancer treatment outcomes. Here, we summarize the genetic changes in human PBMCs reported in previous work and confirm the feasibility of this new tumor screening project. The tumor types in this study were limited to solid tumors because hematological tumors, such as leukemia and lymphoma, have a certain impact on human peripheral blood mononuclear cell gene expression. The purpose of our study was to identify potential biomarkers of cancer at an early stage. Cases of advanced cancer were not included. Through this study, we hope to find a new approach for the development of a blood-based gene expression test for early cancer detection.

## Materials and methods

### Selection of microarray datasets for meta-analysis

We performed a detailed and comprehensive search of microarray datasets in the Gene Expression Omnibus (GEO) database of the National Center for Biotechnology Information (NCBI) (http://www.ncbi.nlm.nih.gov/geo/) according to the Preferred Reporting Items for Systematic Reviews and Meta-Analyses (PRISMA) guidelines published in 2009. To maintain objectivity, the data were extracted from the original database search by two independent reviewers, and any discrepancies between the two reviewers were resolved through consultation with a third reviewer. We used the terms "tumor" and "peripheral blood" as the search keywords for this study, and 797 datasets were obtained from the GEO database. We also included datasets containing the following: 1) RNA research; 2) samples from solid tumors; 3) samples not receiving any tumor treatment; and 4) control samples from the peripheral blood of healthy people. We excluded datasets if they contained the following: 1) hematological tumors, lymphomas, and other tumors that can directly affect related genes in peripheral blood; 2) sample sizes less than 10; 3) terminal cancer samples and 4) nonhuman omics studies.

### Selection of reported genes

We then performed searches in the PubMed database based on the keywords "tumor" and/or "peripheral blood" to explore published articles. We collated articles using the same inclusion and exclusion criteria as for the GEO analysis. After reading the selected literature, we selected relevant reported genes and generated a table that was used in the meta-analysis to identify common genes. Kyoto Encyclopedia of Genes and Genomes (KEGG) pathway enrichment analysis of these genes was also performed to determine relevant biological functions.

### Meta-analysis of microarray datasets

Prior to the meta-analysis, we performed data normalization of the selected datasets using R statistical software (http://www.r-project.org/) to obtain common genes. We summarized the reported genes from the selected datasets, processed the meta-analysis using genes from the normalized datasets and reported genes by using the MAMA, RankProd, affyPLM and CLL software packages in R statistical software. A list of differentially expressed genes (DEGs) was identified based on P-values, and the top ten genes with their corresponding absolute value of P were selected for forest plot analysis to observe differential expression reported in the literature.

### GO annotations and KEGG pathway enrichment analysis

Based on the results of the meta-analysis, the most significant DEGs were evaluated by enrichment analyses. Gene Ontology (GO) annotation and KEGG pathway enrichment analyses were conducted to identify the most significant DEGs using the WEB-based GEne SeT AnaLysis Toolkit (http://www.webgestalt.org/option.php) with a significance threshold of false-discovery rate (FDR) less than 0.1.

### Protein-protein interaction (PPI) network construction

To obtain a clear understanding of the cellular functions and biological activities of PBMC marker genes in solid tumors, we analyzed the DEGs in the Search Tool for the Retrieval of Interacting Genes/Proteins (STRING, http://string-db.org) database. PPI networks were constructed with a confidence score greater than 0.4 as the significance cutoff value, aiming to offer an overview of the functional protein association networks. Afterward, the acquired data were visualized using Cytoscape software.

### Selection of hub genes and modules

CentiScaPe 2.1 was employed to calculate the degree, closeness, and betweenness of the PPI network. We identified hub genes based on the degree of the node. The most important PPI network clustering module was selected in Cytoscape using MCODE software with degree cutoff = 2, node score cutoff = 0.2, k-core = 2, and max depth = 100. The DEGs in each module with an FDR of less than 0.01 were then subjected to pathway enrichment analysis (using STRING) to explore the biological function of each module.

## Results

### Selecting a microarray dataset associated with solid tumors for meta-analysis

**Fig 1:**
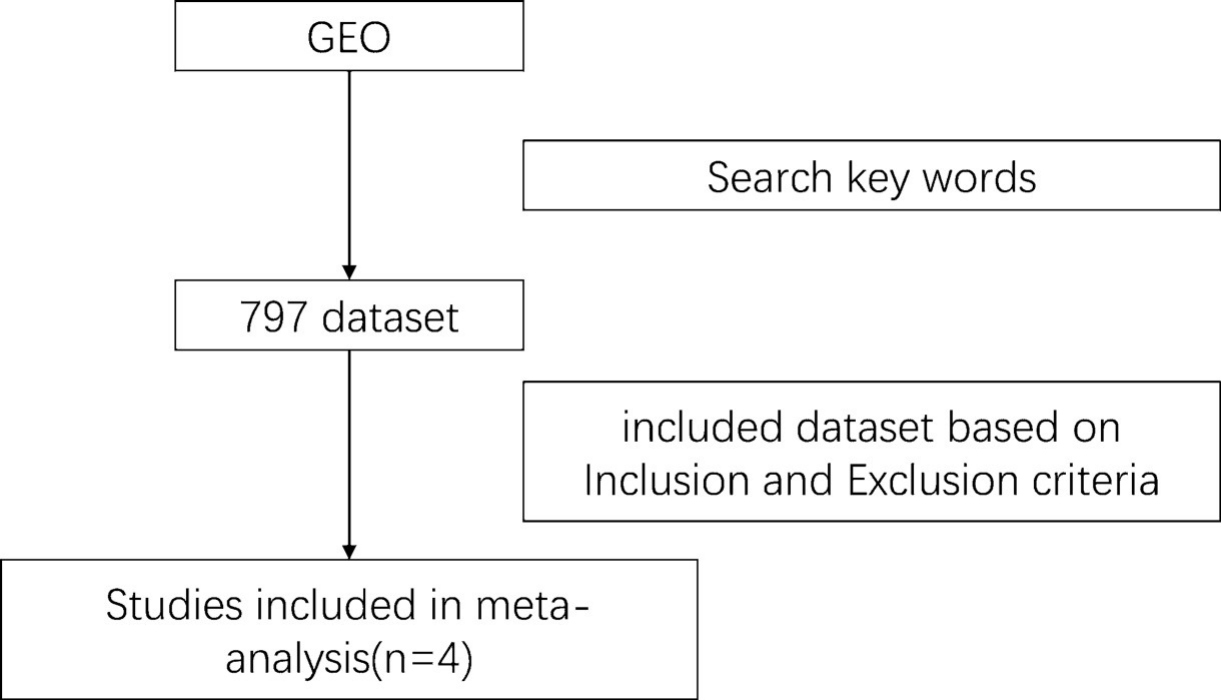
Flow chart of the processing of microarray dataset selection.

**Fig 2:**
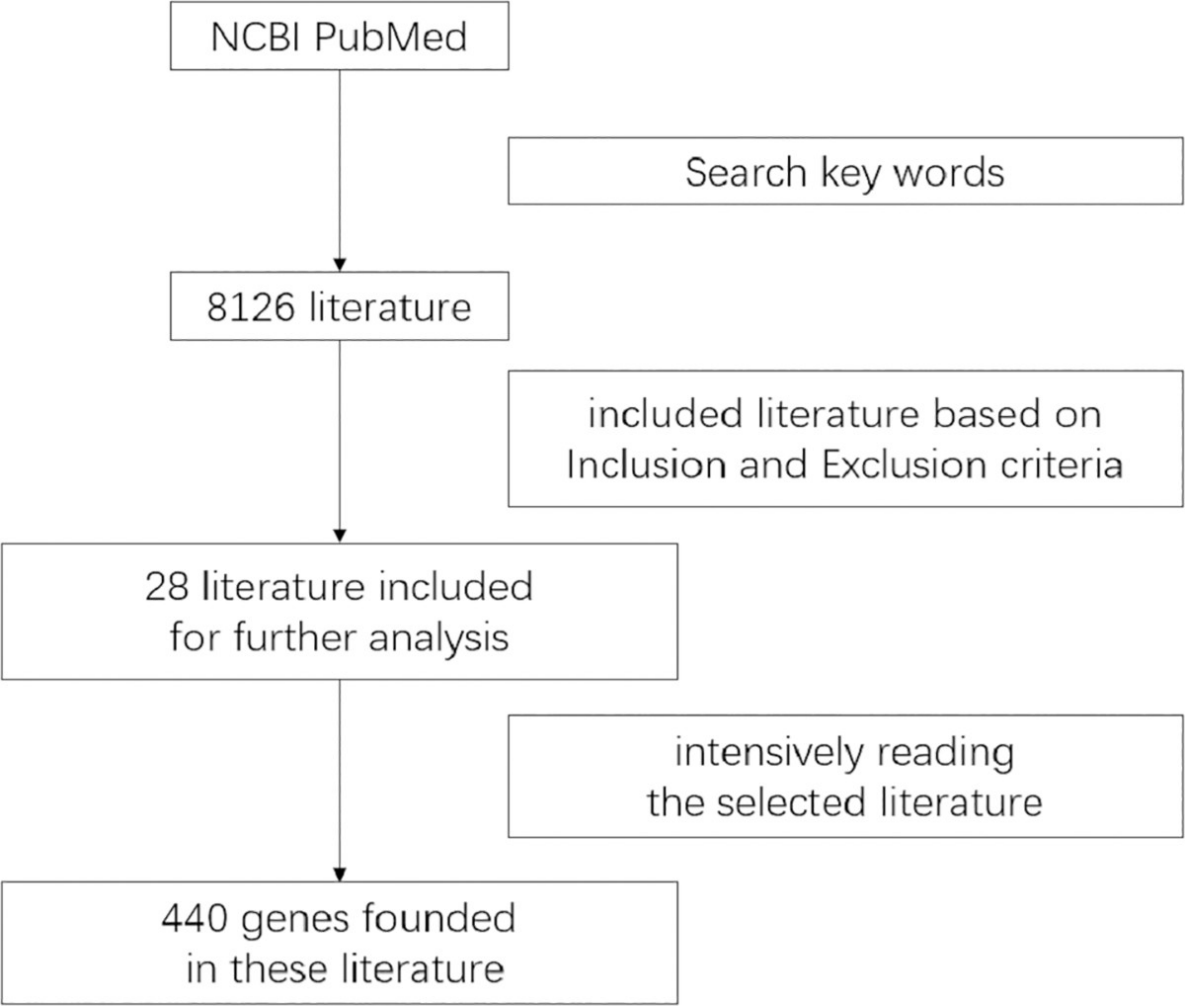
Flow chart of the processing of the reported genes selection.

**Fig 3:**
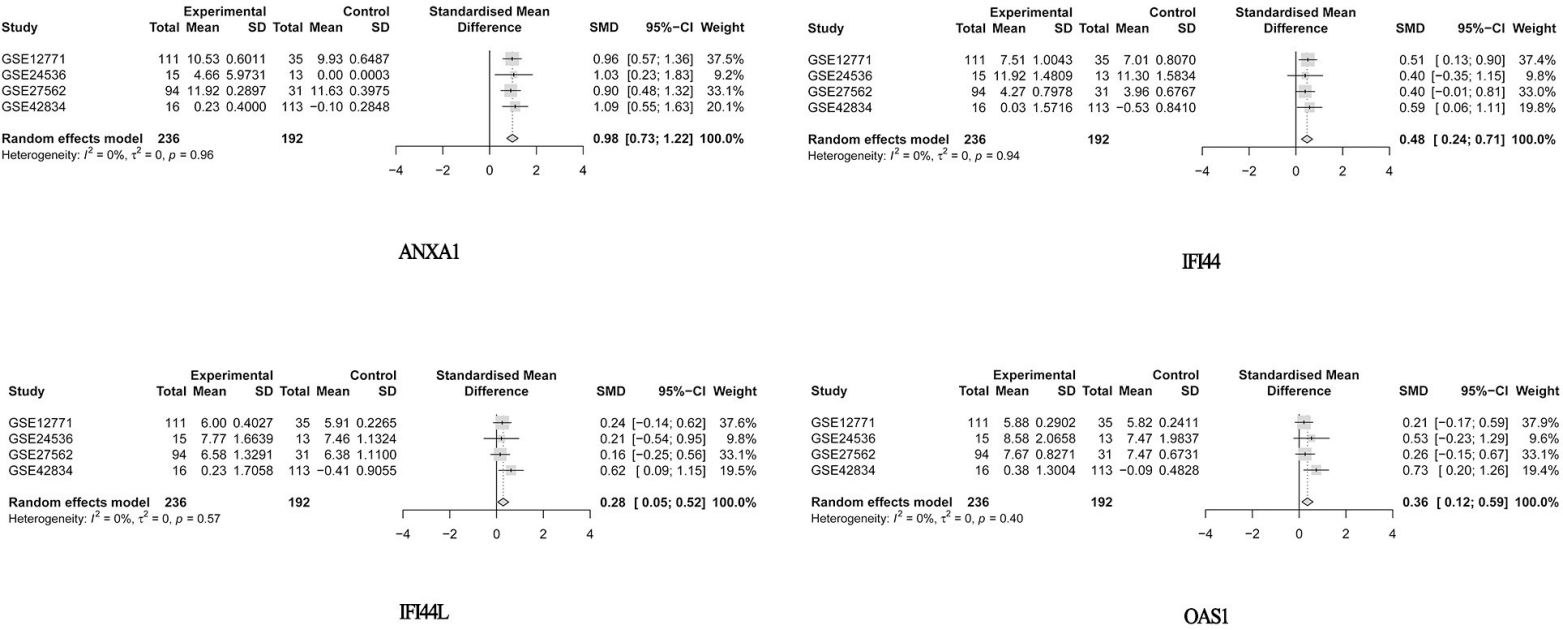
Forest plots of the differential expression levels of the top 4 genes (*ANAX1*, *IFI44*, *IF44L*, and *OAS1*).

According to the inclusion criteria, four datasets (GSE12771, GSE24536, GSE27562, and GSE42834) including 428 samples, 236 solid tumor patients, and 192 healthy controls were chosen from among 797 datasets (see Materials and Methods and Fig 1). The tumor samples consisted of various types of tumors, including breast cancer, lung cancer, and melanoma. The four GEO series (GSE) all used the microarray dataset from tumor samples and normal control samples (Table 1). We retrieved 8,126 related articles from the PubMed database, which were narrowed down to 28 by applying the inclusion and exclusion criteria. A total of 440 reported genes were found in these articles (S1 Table); the flow chart is shown in Fig 2. As some genes appeared only once, the results were not stable. In the meta-analysis, 285 genes from among 440 reported genes were selected using the MAMA, RankProd, affyPLM and CLL software packages in the R statistical software. (S2 Table), and 20 genes, the top ten with positive and negative absolute P-values were used in forest plot analysis (S1 and S2 Files).Ultimately, 4 genes (*ANXA1*, *IFI44*, *IFI44L*, and *OAS1*) with significant forest plots were determined as the marker genes of PBMCs (Fig 3).

**Table 1:** Basic information of the four GSE datasets.

study	sample	case/control	conutry	PMID	platform	Cancer type
GSE24536	28	15/13	USA	21555851	GPL6480	Melanoma
GSE27562	125	94/31	USA	21781289	GPL570	Breast Tumor
GSE42834	129	16/113	England	23940611	GPL10558	Lung Cancer
GSE12771	146	111/35	Germany	21558400	GPL6102	Lung Cancer

### GO and KEGG pathway enrichment analysis

**Fig 4:**
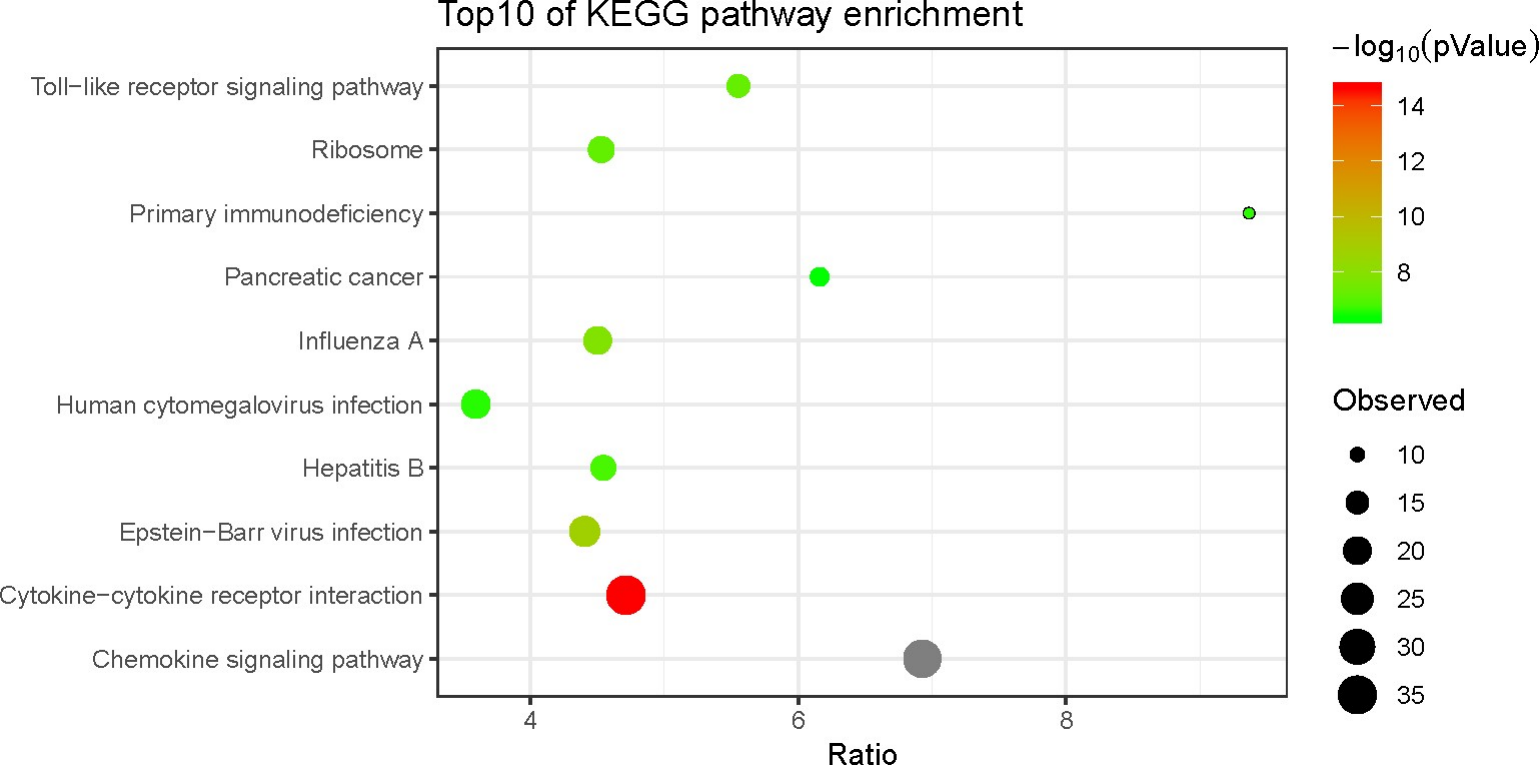
The top 10 KEGG enrichment pathways of DEGs.

To further investigate the function of the reported genes and DEGs, we performed a KEGG pathway enrichment analysis of 440 genes with a significance threshold of less than 0.1 (Table 2). We also performed biological process functional GO and KEGG pathway enrichment analyses on 285 genes (Table 3) and constructed a channel-enriched bubble chart of the KEGG pathway enrichment analysis using the ggplot2 software package in R software (Fig 4). The bubble chart shows the ratio on the horizontal axis and the path name on the vertical axis, and the size of the bubble represents the observed gene number. The color of the bubble represents the -log10 P-value. Therefore, larger bubbles indicate the detection of more genes in the pathway, and the deeper the bubble color is, the smaller the P-value of the pathway is. We identified two significant pathways and the ‘primary immunodeficiency’ pathway and ‘cytokine-cytokine receptor interaction’ pathway, through this analysis.

**Table 2:** KEGG pathway enrichment analysis of 440 genes.

Gene Set	Description	Size	Overlap	Expected	Enrichment Ratio	P Value	FDR
hsa04062	Chemokine signaling pathway	189	34	5.086223	6.684724526	0	0
hsa04060	Cytokine-cytokine receptor interaction	294	36	7.911903	4.55010661	7.99E-15	1.30E-12
hsa03010	Ribosome	153	21	4.117419	5.100282899	5.91E-10	6.42E-08
hsa05169	Epstein-Barr virus infection	201	23	5.409158	4.252048217	3.33E-09	2.72E-07
hsa05164	Influenza A	171	20	4.601821	4.346105729	2.59E-08	1.69E-06
hsa04620	Toll-like receptor signaling pathway	104	15	2.798768	5.359500574	9.67E-08	5.25E-06
hsa05340	Primary immunodeficiency	37	9	0.995716	9.038725292	4.01E-07	1.87E-05
hsa05161	Hepatitis B	144	16	3.875218	4.128800442	1.38E-06	5.63E-05
hsa05160	Hepatitis C	131	15	3.525372	4.254870685	2.04E-06	6.89E-05
hsa05162	Measles	132	15	3.552283	4.222636816	2.25E-06	6.89E-05

**Table 3:** GO and KEGG pathway enrichment analysis of 285 genes.

Category	Geneset	Description	Size	Overlap	Expected	Enrichment Ratio	P Value	FDR
GO	GO:0002521	leukocyte differentiation	496	42	9.043149436	4.644399642	0	0
	GO:0002694	regulation of leukocyte activation	481	42	8.769667094	4.789235389	0	0
	GO:0098542	defense response to other organism	473	43	8.623809845	4.986195286	0	0
	GO:0042110	T cell activation	452	40	8.240934567	4.85381842	0	0
	GO:0050900	leukocyte migration	419	40	7.639273415	5.236100062	0	0
	GO:1903706	regulation of hemopoiesis	389	40	7.092308731	5.639912406	0	0
	GO:0009615	response to virus	319	37	5.816057803	6.361697434	0	0
	GO:0060326	cell chemotaxis	289	34	5.269093119	6.452723312	0	0
	GO:0070661	leukocyte proliferation	281	35	5.12323587	6.831619876	0	0
	GO:1990868	response to chemokine	93	24	1.695590519	14.15436081	0	0
KEGG	hsa04062	Chemokine signaling pathway	189	34	4.909090909	6.925925926	0	0
	hsa04060	Cytokine-cytokine receptor interaction	294	36	7.636363636	4.714285714	2.44249E-15	3.98126E-13
	hsa05169	Epstein-Barr virus infection	201	23	5.220779221	4.405472637	1.65478E-09	1.7982E-07
	hsa05164	Influenza A	171	20	4.441558442	4.502923977	1.41264E-08	1.1513E-06
	hsa04620	Toll-like receptor signaling pathway	104	15	2.701298701	5.552884615	6.03174E-08	3.86041E-06
	hsa03010	Ribosome	153	18	3.974025974	4.529411765	7.10504E-08	3.86041E-06
	hsa05161	Hepatitis B	144	17	3.74025974	4.545138889	1.5876E-07	7.39366E-06
	hsa05340	Primary immunodeficiency	37	9	0.961038961	9.364864865	2.96446E-07	1.13916E-05
	hsa05163	Human cytomegalovirus infection	225	21	5.844155844	3.593333333	3.14492E-07	1.13916E-05
	hsa05212	Pancreatic cancer	75	12	1.948051948	6.16	4.18383E-07	1.36393E-05

### Hub gene and module screening from the PPI network

**Fig 5:**
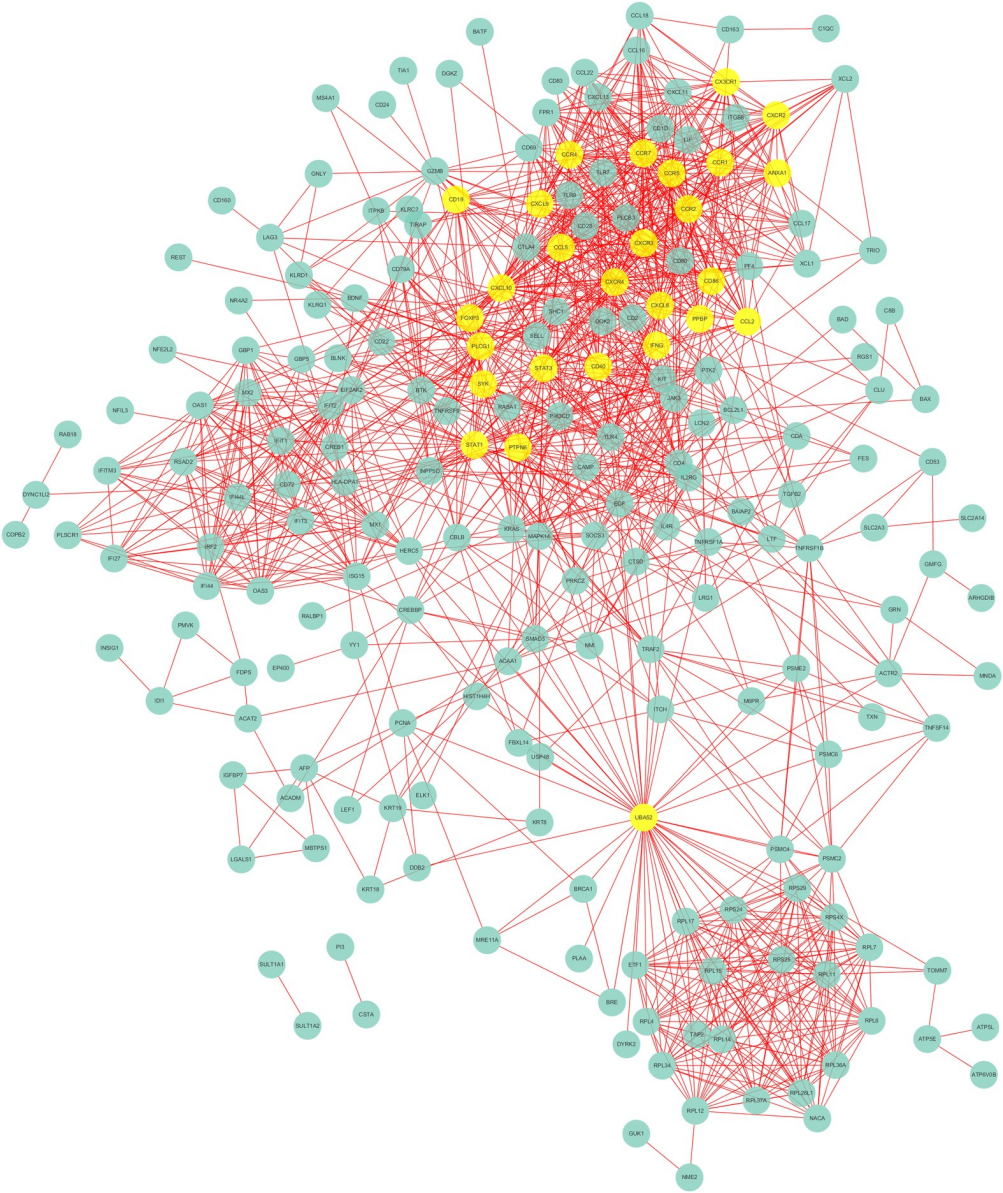
Hub genes acquired from the PPI network.

**Fig 6:**
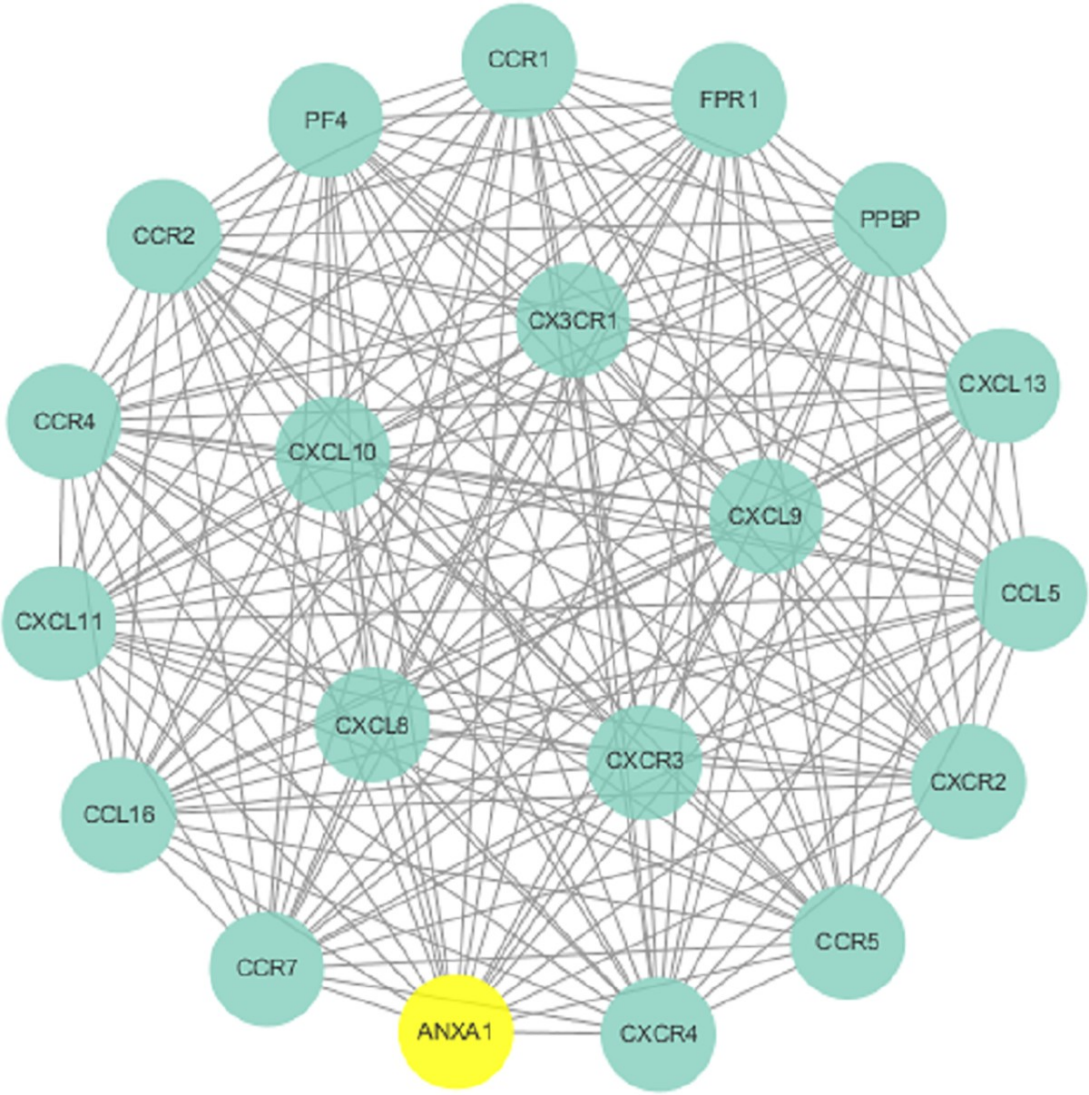
Cluster 1 selected from the PPI network.

**Fig 7:**
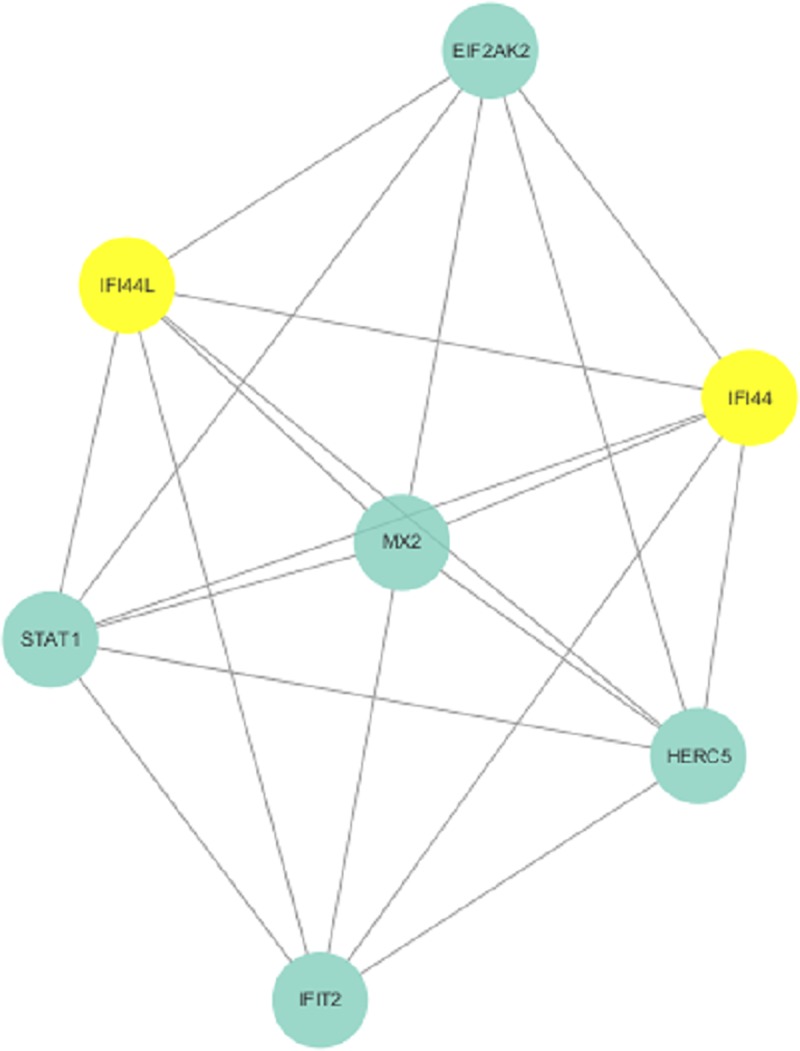
Cluster 2 selected from the PPI network.

**Fig 8:**
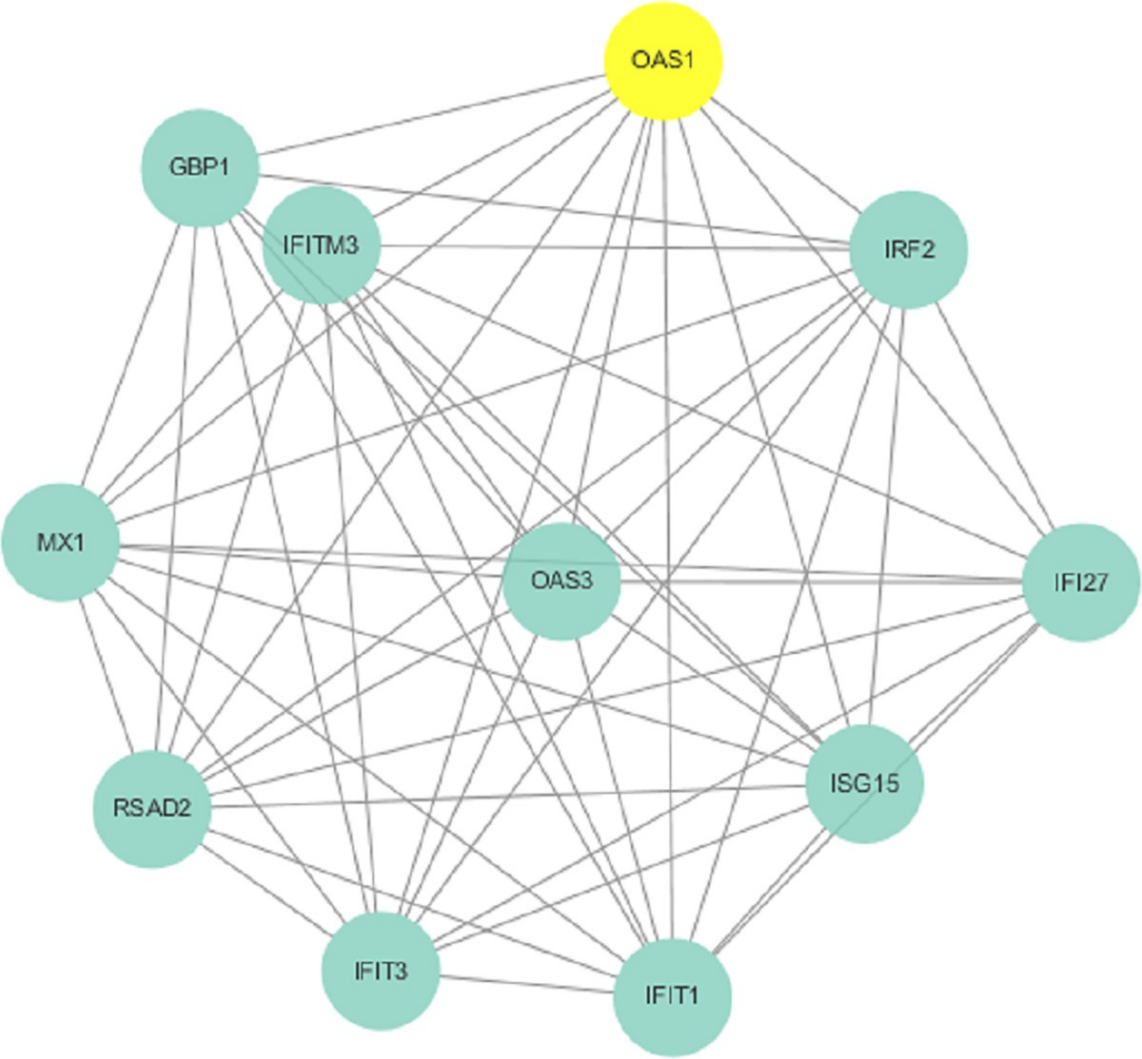
Cluster 3 selected from the PPI network.

To further visualize the cellular functions and biological activities of the 285 genes, a genetic interaction network map of DEGs was drawn in Cytoscape. First, we identified a PPI network, which consisted of 285 nodes and 2,655 edges with a confidence score greater than 0.4 based on the STRING database. The top 27 hubs with degree centrality greater than 23 were screened as hub genes from the PPI network. These genes included annexin A1 (*ANXA1*), signal transducer and activator of transcription 1 (*STAT1*), signal transducer and activator of transcription 3 (*STAT3*), CX3C motif chemokine receptor 1 (*CX3CR1*), CXC motif chemokine receptor 2 (*CXCR2*), CC motif chemokine receptor 1 (*CCR1*), CC motif chemokine receptor 5 (*CCR5*), CC motif chemokine receptor 7 (*CCR7*), CC motif chemokine receptor 4 (*CCR4*), CC motif chemokine receptor 2 (*CCR2*), CXC motif chemokine ligand 9 (*CXCL9*), CD19 molecule (*CD19*), CXC motif chemokine receptor 3 (*CXCR3*), C motif chemokine ligand 5 (*CCL5*), CXC motif chemokine receptor 4 (*CXCR4*), CD86 molecule (*CD86*), CXC motif chemokine ligand 10 (*CXCL10*), forkhead box P3 (*FOXP3*), CXC motif chemokine ligand 8 (*CXCL8*), pro-platelet basic protein (*PPBP*), CC motif chemokine ligand 2 (*CCL2*), interferon gamma (*IFNG*), phospholipase C gamma 1 (*PLCG1*), spleen associated tyrosine kinase (*SYK*), CD40 molecule (*CD40*), protein tyrosine phosphatase nonreceptor type 6 (*PTPN6*), and ubiquitin A-52 residue ribosomal protein fusion product 1 (*UBA52*) (Fig 5). We then found 3 modules in the MCODE analysis and used Cluster 1 (related to *ANXA1*), Cluster 2 (related to *IFI44* and *IFI44L*) and Cluster 3 (related to *OAS1*) for KEGG pathway enrichment analysis (Figs 6–8, Tables 4, S3 and S4).

**Table 4:** The KEGG pathway enrichment analysis of Cluster 1.

Geneset	Description	Size	Overlap	Expected	Enrichment Ratio	P Value	FDR
hsa04060	Cytokine-cytokine receptor interaction	294	18	0.74789128	24.0676692	0	0
hsa04062	Chemokine signaling pathway	189	18	0.48078725	37.4385965	0	0
hsa04620	Toll-like receptor signaling pathway	104	5	0.26456018	18.8992915	4.73E-06	5.14E-04
hsa05163	Human cytomegalovirus infection	225	6	0.57236578	10.482807	1.37E-05	0.00111274
hsa05120	Epithelial cell signaling in Helicobacter pylori infection	68	3	0.17298166	17.3428793	6.30E-04	0.04108467
hsa05167	Kaposi sarcoma-associated herpesvirus infection	186	4	0.47315571	8.45387663	0.00107644	0.05848636
hsa05164	Influenza A	171	3	0.43499799	6.89658356	0.0087241	0.40629393
hsa04623	Cytosolic DNA-sensing pathway	63	2	0.16026242	12.4795322	0.01091775	0.44489832
hsa04622	RIG-I-like receptor signaling pathway	70	2	0.17806935	11.2315789	0.01335851	0.48387498
hsa05323	Rheumatoid arthritis	90	2	0.22894631	8.73567251	0.02149547	0.62095381
hsa04144	Endocytosis	244	3	0.62069889	4.83326143	0.02266138	0.62095381
hsa04657	IL-17 signaling pathway	93	2	0.23657786	8.45387663	0.0228572	0.62095381
hsa05142	Chagas disease (American trypanosomiasis)	102	2	0.25947249	7.70794634	0.02715131	0.6808712
hsa04668	TNF signaling pathway	110	2	0.27982327	7.14736842	0.03122221	0.72703143

## Discussion

The current early screening tools for tumors are limited to use at an identifiable stage, and most are invasive. The early stage of tumor formation may change the immune components in human PBMCs; Thus, utilizing such changes will aid in the detection of a new target for the early screening of tumors. Because tumors caused by blood diseases such as leukemia may change the composition of human PBMCs without an immune response, this study focused on early screening of solid tumors only. In addition, the statistics of terminal stages of cancer are not conducive to establishing a biomarker indicating the transformation of early stages. Ultimately, we examined 4 datasets chosen from among 797 datasets, involving 3 types of solid cancer.

In this study, we performed a meta-analysis of PBMCs between patients with solid tumors and normal healthy individuals to define possible target genes. A total of 285 genes were selected during this analysis. According to forest plot analysis of the 20 top genes, 4 genes (*ANXA1*, *IFI44*, *IFI44L*, and *OAS1*) were determined as the marker genes of PBMCs. All the P-values of the 4 genes were significant (P>0.05), demonstrating the stability of the genes being presented across each of the datasets. To classify the function of the 285 genes, GO and KEGG pathway enrichment analyses were performed. Furthermore, we chose the top 27 hub nodes with a degree centrality greater than 23 from the PPI network as hub genes and found 3 important clusters related to the four selected genes (*ANXA1*, *IFI44*, *IFI44L*, and *OAS1*). KEGG pathway enrichment analysis was also used to investigate the functions of these modules.

As reported in the majority of previous studies, *ANXA1* is produced by many cell types, including peripheral blood leukocytes, where *ANXA1* is mainly expressed in neutrophils.[17] Loss of function or expression of *ANXA1* has been detected in multiple tumors. *ANXA1* may function as either a tumor suppressor or a tumor promoter, depending on the type of tumor cells/tissues [18]. Additionally, some studies have shown that its positive expression is correlated positively with the progression of several types of cancers. In our study, we found that *ANXA1* was overexpressed in the PBMCs of cancer patients, and thus hypothesized that it could serve as a biomarker of cancer diagnosis.

In terms of our analysis, we found the highest ratio of *ANXA1* in the primary immunodeficiency pathway. Defects in immune cells, such as suppression of immune cell proliferation in patients, may be diagnosed as primary immunodeficiency. Enhanced expression of *ANXA1* might reduce the *in vitro* peripheral blood lymphocyte response to mitogens, activate the ERK/MAPK pathway and reduce immune cell proliferation by disrupting the actin skeleton and abolishing cyclin D1 expression[19]. All these events give rise to primary immunodeficiency, facilitating tumor immunity escape. Other studies have reported the possible effects of *ANXA1* on mitogen-activated T cells in humans. Consequently, overexpression of *ANXA1* results in malignant proliferation of cancer cells by causing disorder in the immune system. Moreover, it was reported that increased *ANXA1* expression can abolish *COX-2* expression. *COX-2* exerts a negative effect on immune surveillance, plays a key role in tumorigenesis, and is associated with angiogenesis in the transition period of carcinoma [20]. These mechanisms indicate that *ANXA1* can mediate many diverse cellular functions, such as inflammation and proliferation, and has an important effect on suppressing the development of cancer.

In addition to the primary immunodeficiency pathway, a large proportion of the summarized genes, including *ANXA1*, are involved in the cytokine-cytokine receptor interaction reference pathway that affects the status of carcinoma. Most genes in Cluster 1 are linked to the cytokine-cytokine receptor interaction. Moreover, *ANXA1* was included in Cluster 1, with many genes included in the CC and CXC subfamilies. Both families of chemokine factors are involved in the chemotaxis of leukocytes and promote the proliferation of immune cells, resulting in pleiotropic effects including the stimulation of monocytes, natural killer and T-cell migration, and the modulation of adhesion molecule expression. These actions inhibit the expansion of tumors. Thus, we suggest that *ANXA1* might influence the course of neoplasms by affecting interaction between cytokines. High *ANXA1* expression exerts its effect via inhibition of CC and CXC subfamily members, leading to a restricted in immune response to cancers. Regarding other aspects of the cytokine-cytokine receptor interaction reference pathway, cytoplasmic *ANXA1* exhibits anti-inflammatory activity by inhibiting phospholipase A2. Extracellular *ANXA1* regulates leukocyte migratory events through interactions with n-formyl peptide receptors, binding to the formyl peptide receptor (FPR) on neutrophils and preventing transendothelial extravasation. These activities interrupt the process of leukocyte migratory events and suppress immune system attack of cancers. These findings may explain elevated expression of *ANXA1* in infiltrating leukocytes. Alternately, as a substrate protein of EGFR, *ANXA1* may contribute to neoplasm growth via autocrine and paracrine effects and sustain the preinvasive properties of malignant cancers through autocrine signaling induced by the N-terminal peptide [21].

All the samples we selected were from cancer patients with an exact diagnosis, most reaching a diagnosable stage. At this stage, the immune system is weaker with regard to combating cancer and cannot effectively stop the malignant growth of tumors. *ANXA1* is expressed at low levels in PBMCs with benign tumors, which would affect malignant expansion. Decreased expression of *ANXA1* has been shown to be responsible for a strong delay of proliferation, migration/invasion, and angiogenesis in melanoma, lung carcinoma, NSCLC, breast cancer, and prostate cancer models [22]. In general, *ANXA1* is aberrantly expressed in both benign and malignant tumor stages compared with that in the healthy population. Because of its abnormal expression in PBMCs, *ANXA1* might be a meaningful biomarker for cancer diagnosis and is considered a primary mediator of anti-inflammatory activity.

Our study also found *IFI44*, *IFI44L*, and *OAS1* to be overexpressed. These genes are associated with interferons. Expression of *OAS1* is induced by interferons against cancers. *IFI44* belongs to the INF-α family, mediating the inflammatory response. *IFI44L* might be a novel tumor suppressor that affects cancer stemness, metastasis, and drug resistance in cancer cells.

Radiotherapy and systemic chemotherapy are the traditional choices of treatments for patients with cancer. However, they also induced a range of side effects due to their nonselective killing of malignant and normal cells. Immune checkpoint blockade has improved cancer treatment with lower rates of treatment-related toxicity. Abnormal changes in the molecular characteristics of the immune microenvironment are also helpful for the early diagnosis of malignant tumors and for understanding of immunosuppression in patients. In this study, overexpression of *ANXA1* in the PBMCs of cancer patients, depending on the cancer proliferation status, suggests the potency of *ANXA1* as a biomarker of the identification of cancer. This finding might help us in detecting cancer much earlier. Furthermore, this *ANXA1* overexpression depending on the cancer proliferation status suggests the potency of *ANXA1* as a biomarker of the identification of cancer, aiding in the identification of cancer early.

This study was exploratory, defining a specific tool using PBMCs as biomarker for identifying solid tumors at an early stage. This approach has not yet been popularized and requires formal and independent validation. Nonetheless, we hope to continue this research on the basis of our results.

## Conclusions

*ANXA1* is involved in the immunosuppressive mechanism of tumor-bearing hosts and can be used as a new strategy involving the use of the host's own immunity to achieve tumor suppression. *IFI44*, *IFI44L* and *OAS1* are potential diagnostic biomarkers, though the results for these genes were not as remarkable as those for *ANXA1*. However, our study mainly describes the variation in *ANXA1* in several solid cancers, and the inclusion of more cancer types and application of further experiments for validation are needed.

## Supporting information

S1 Files Forest plot of the differential expression levels of the top ten positive absolute P-value genes. (ZIP)

S2 Files Forest plot of the differential expression levels of the top ten negative absolute P-value genes. (ZIP)

S1 Table The 440 reported genes from the PubMed database. (XLSX)

S2 Table The 285 selected genes from reported genes and common genes. (DOCX)

S3 Table KEGG pathway enrichment analysis of Cluster 2. (DOCX)

S4 Table KEGG pathway enrichment analysis of Cluster 3. (DOCX)
