# Supplementary figures and images for: Identification of human peripheral blood monocyte gene markers for early screening of solid tumors
Source: PLoS One. 2020 Mar 30;15(3):e0230905. doi: 10.1371/journal.pone.0230905 (PMC7105127; doi:10.1371/journal.pone.0230905)

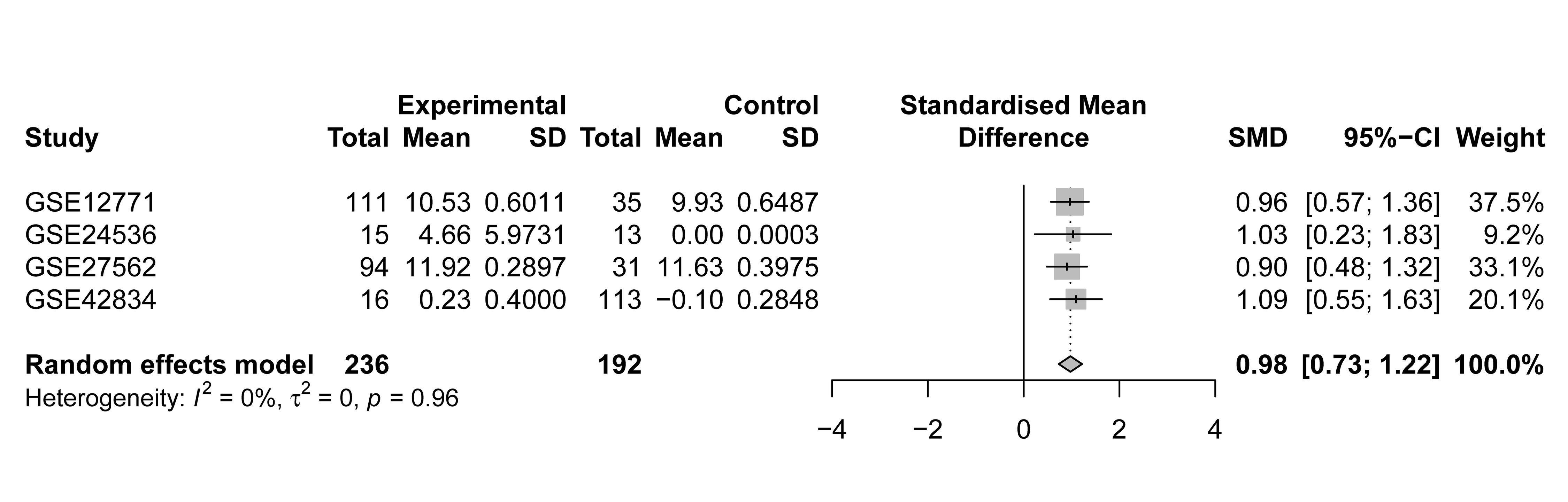

Supplement: S1 Files — (ZIP) [file pone.0230905.s001.zip › S1_File/ANXA1.tif]

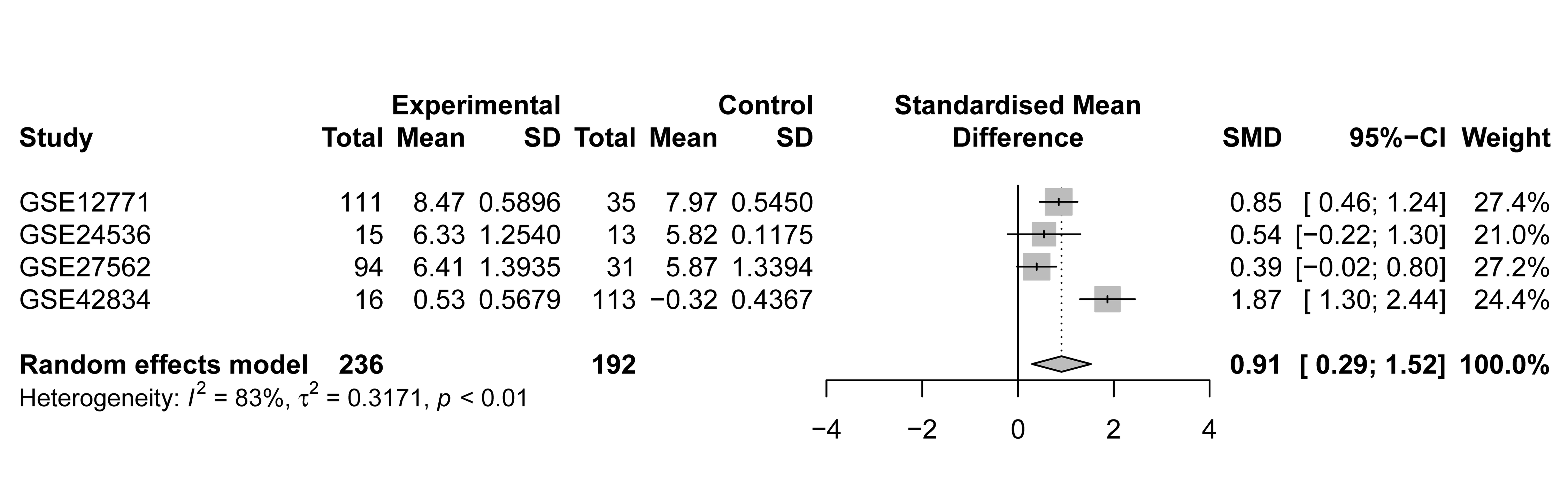

Supplement: S1 Files — (ZIP) [file pone.0230905.s001.zip › S1_File/CCR2.tif]

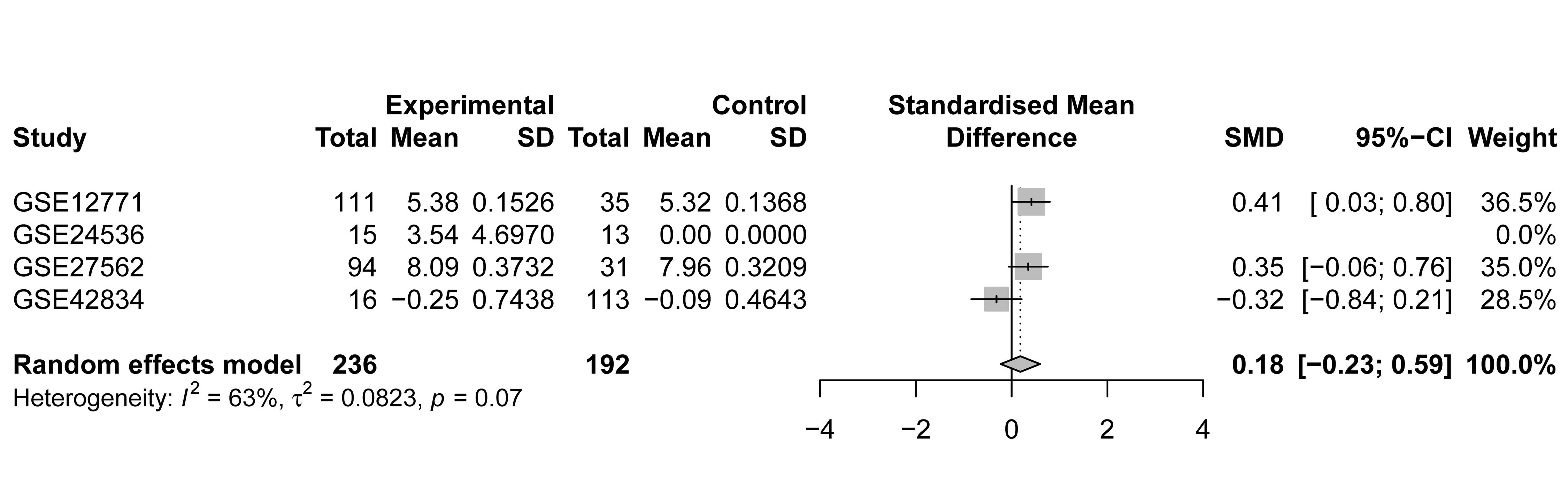

Supplement: S1 Files — (ZIP) [file pone.0230905.s001.zip › S1_File/CHD2.tif]

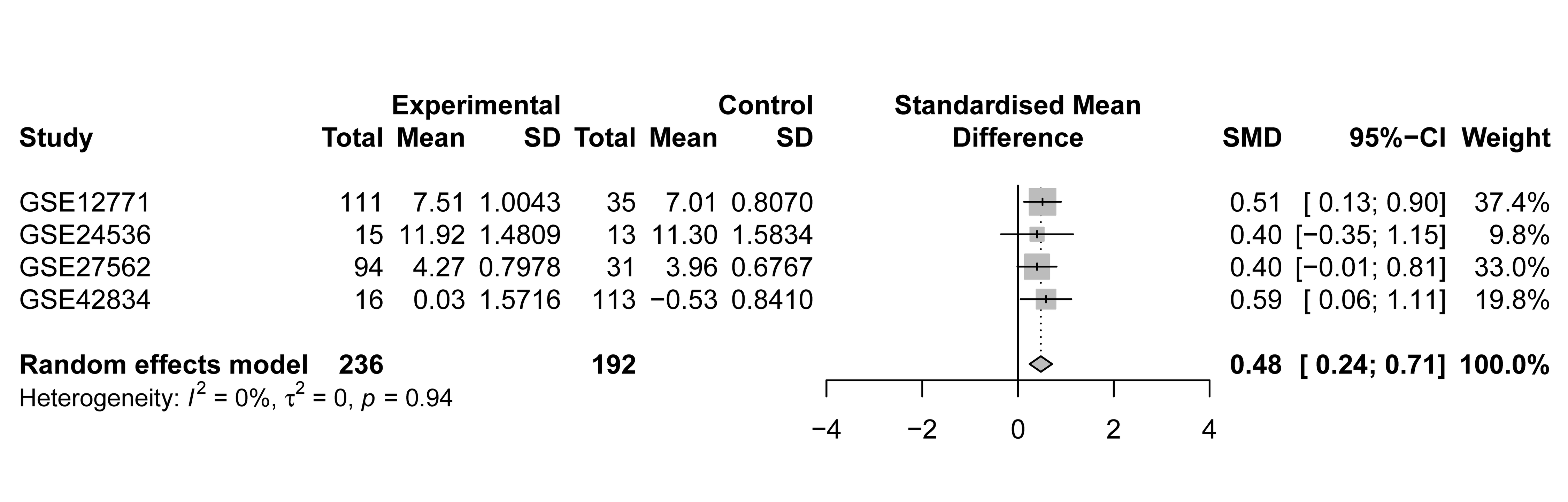

Supplement: S1 Files — (ZIP) [file pone.0230905.s001.zip › S1_File/IFI44.tif]

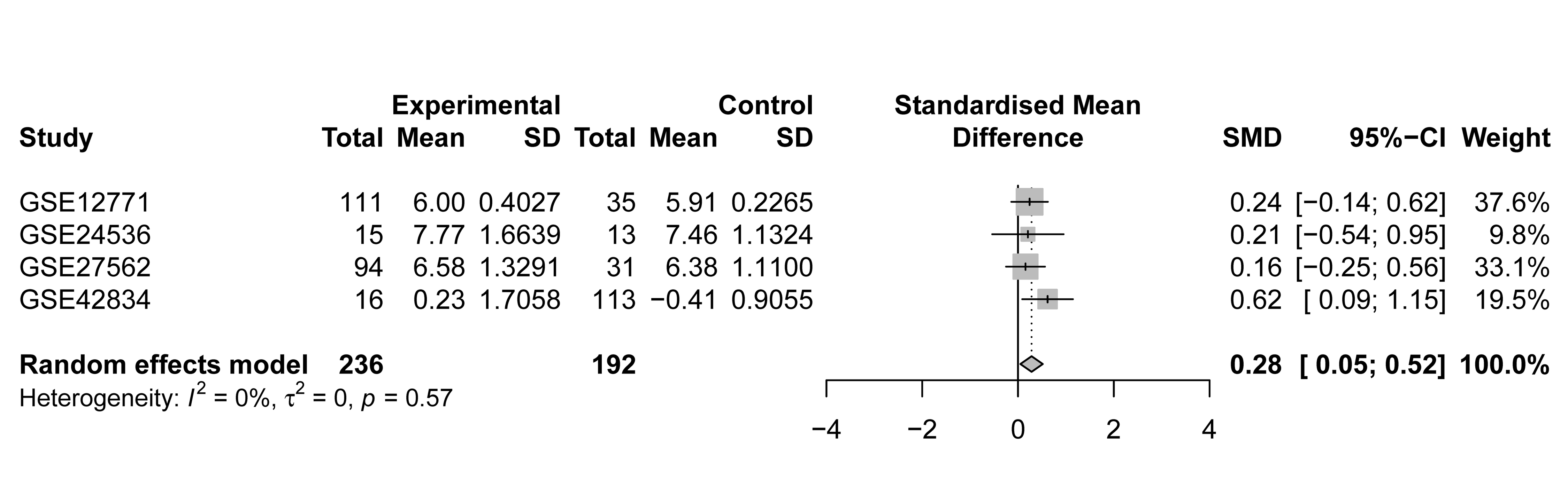

Supplement: S1 Files — (ZIP) [file pone.0230905.s001.zip › S1_File/IFI44L.tif]

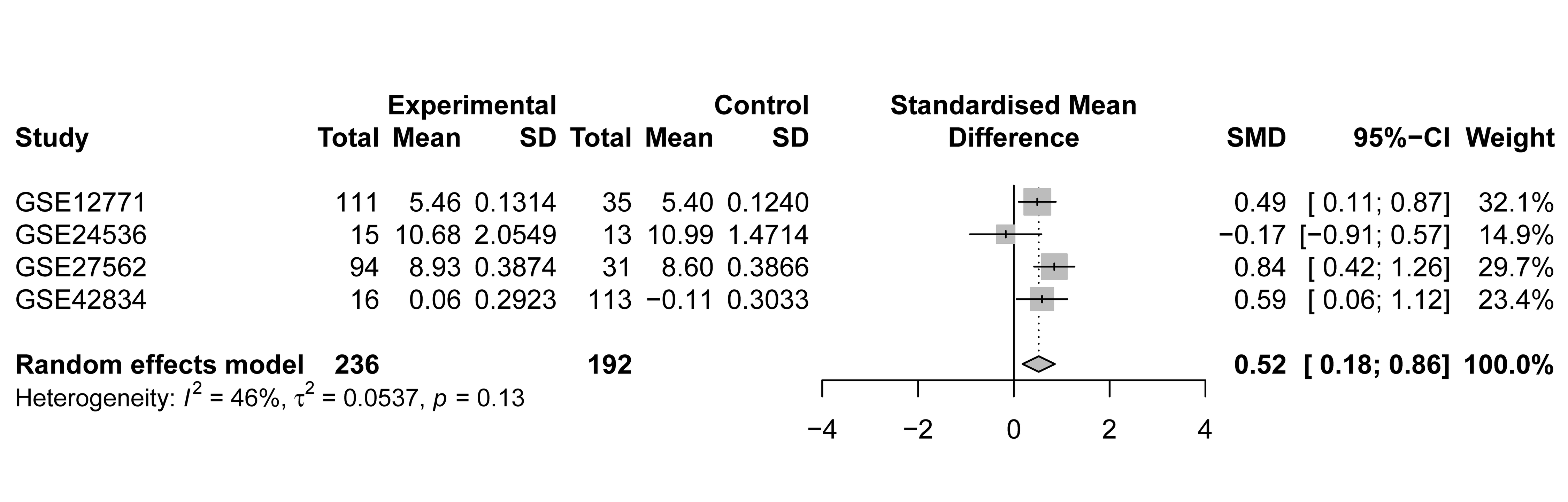

Supplement: S1 Files — (ZIP) [file pone.0230905.s001.zip › S1_File/INPP5D.tif]

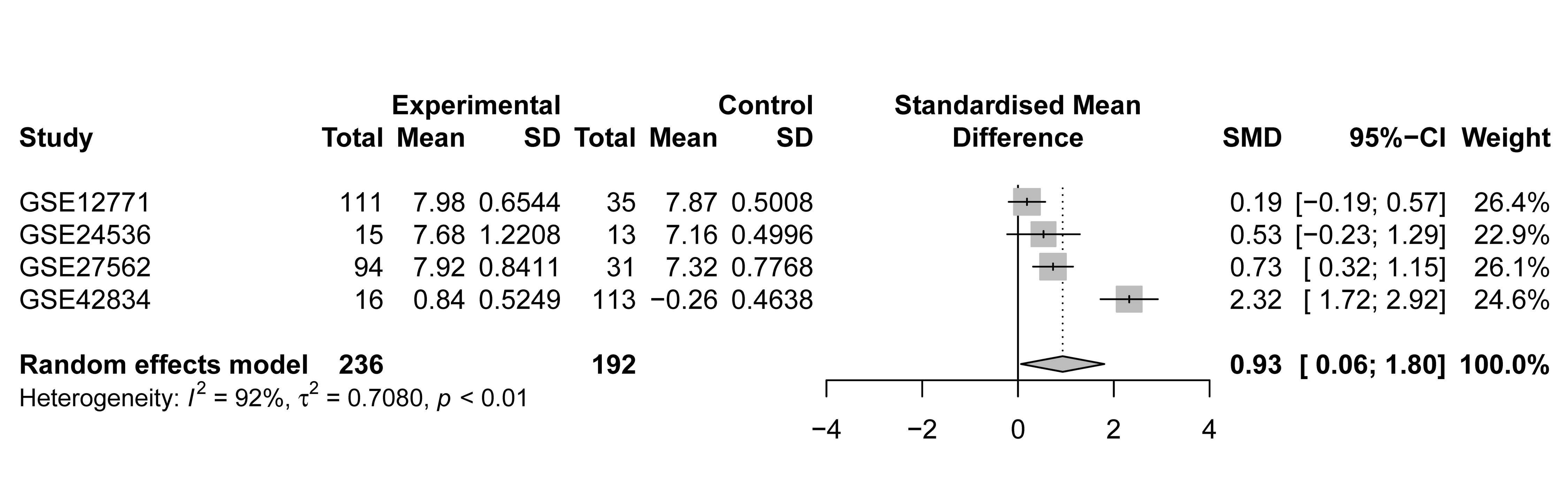

Supplement: S1 Files — (ZIP) [file pone.0230905.s001.zip › S1_File/NFIL3.tif]

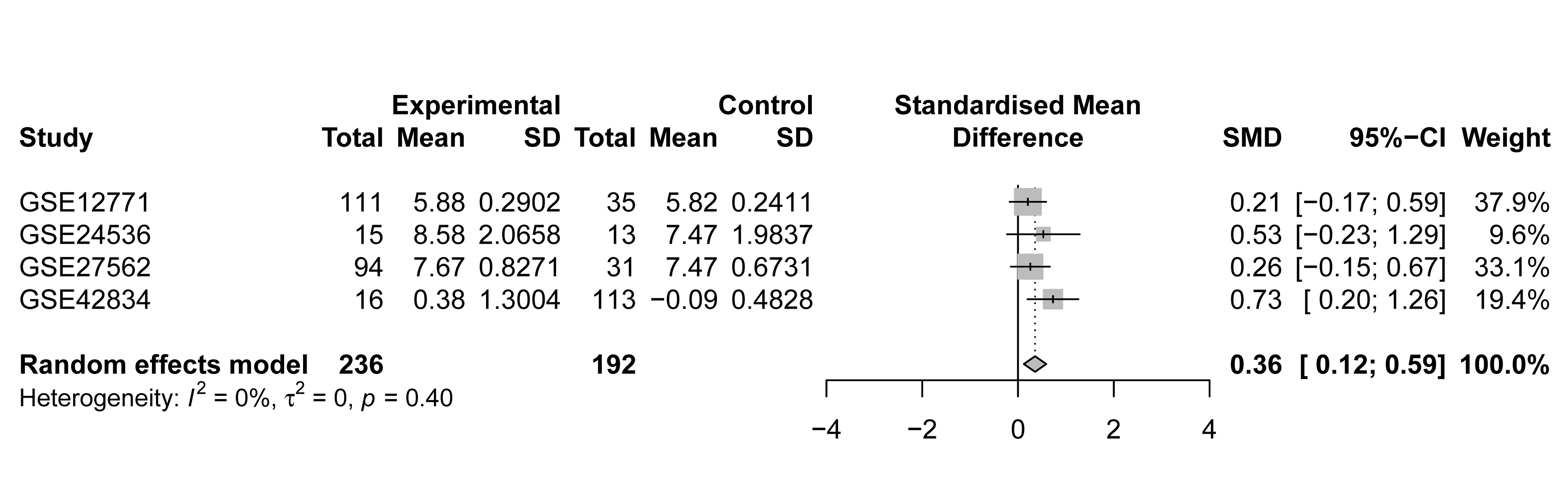

Supplement: S1 Files — (ZIP) [file pone.0230905.s001.zip › S1_File/OAS1.tif]

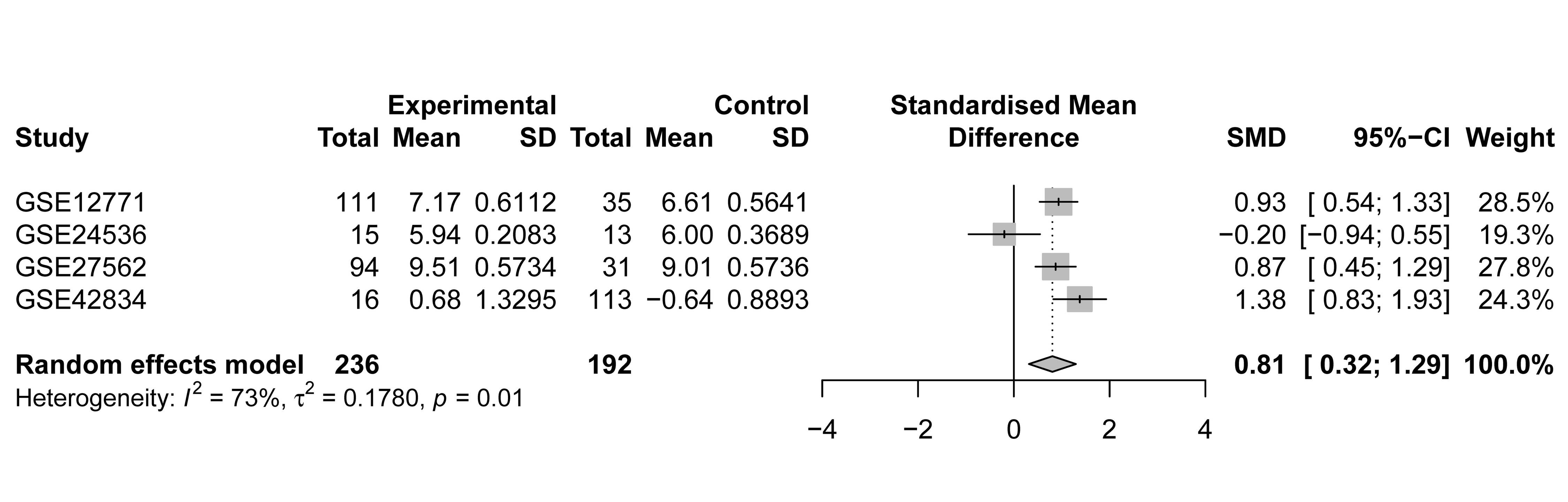

Supplement: S1 Files — (ZIP) [file pone.0230905.s001.zip › S1_File/PLSCR1.tif]

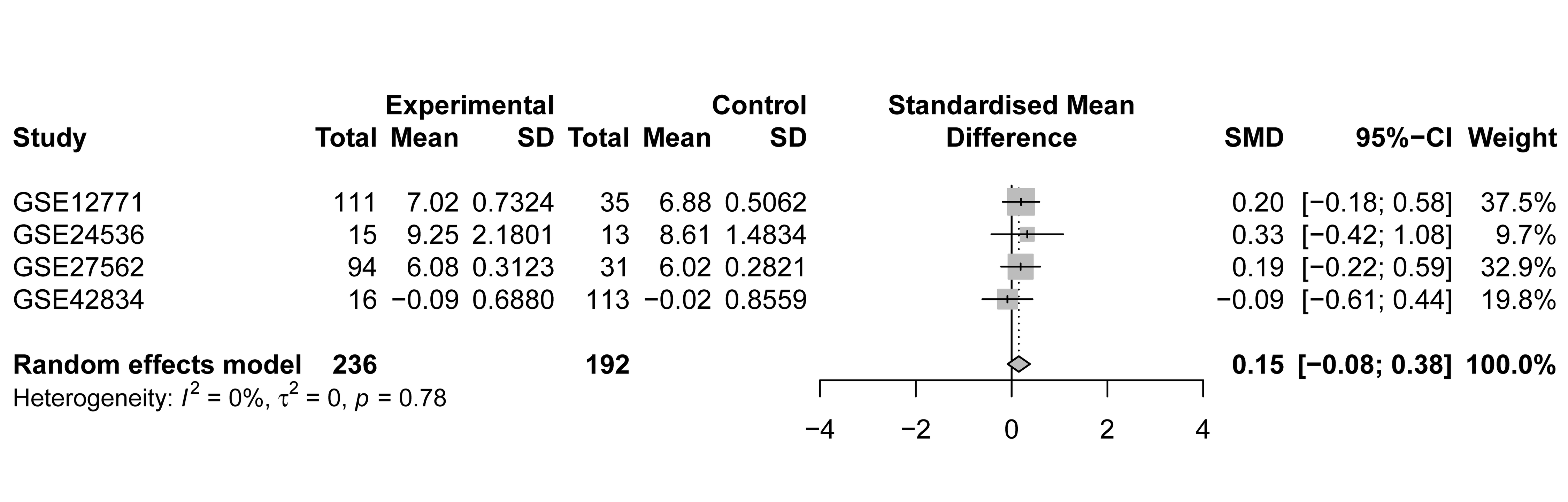

Supplement: S1 Files — (ZIP) [file pone.0230905.s001.zip › S1_File/SLC1A5.tif]

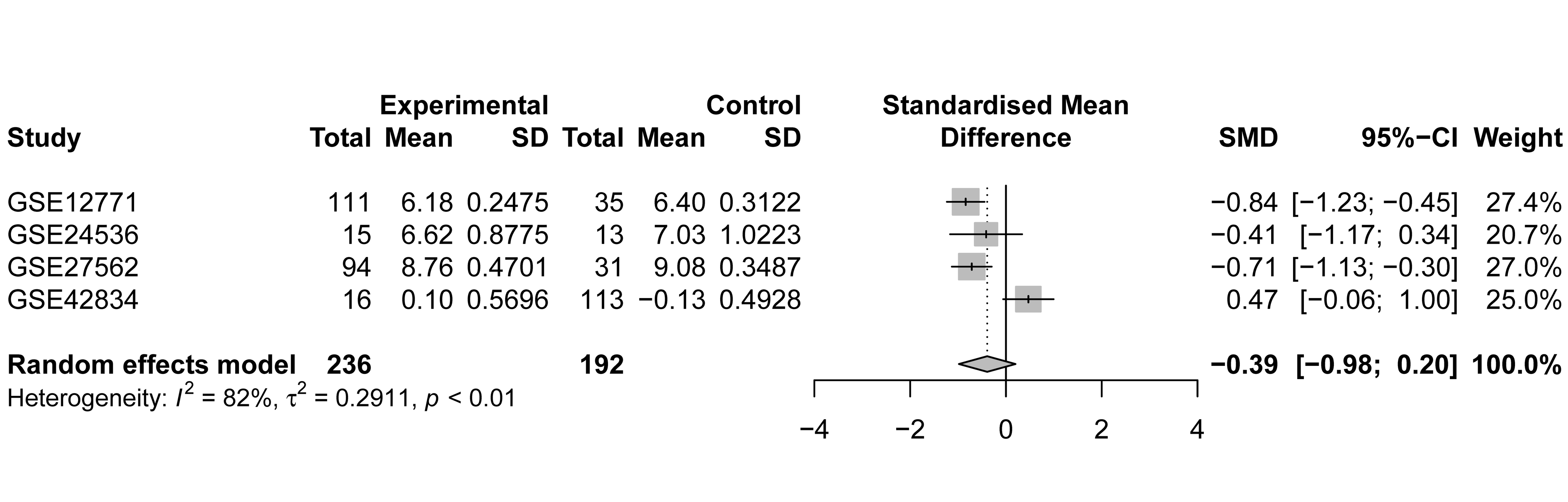

Supplement: S2 Files — (ZIP) [file pone.0230905.s002.zip › S2_File/C9orf64.tif]

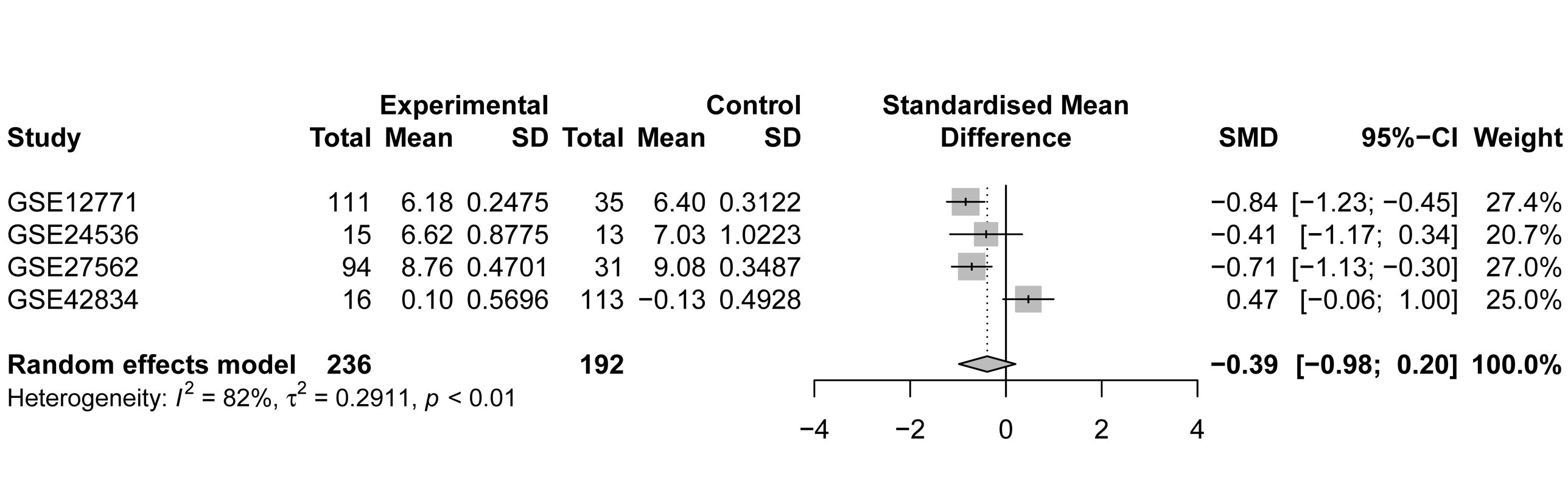

Supplement: S2 Files — (ZIP) [file pone.0230905.s002.zip › S2_File/C9orf64.tiff]

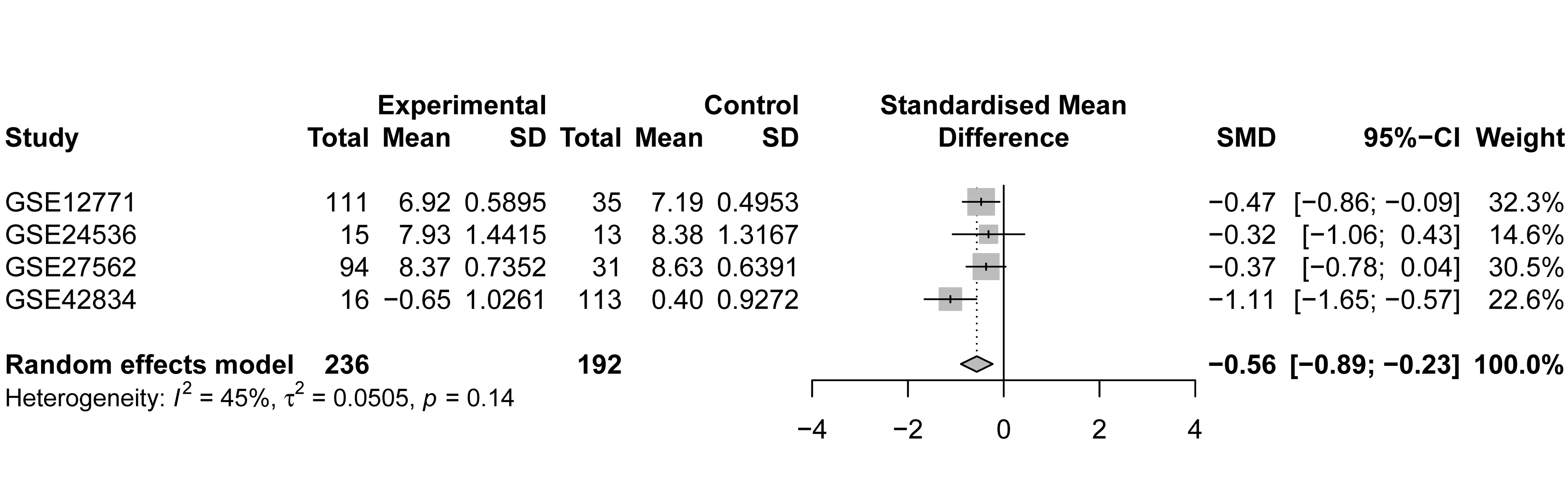

Supplement: S2 Files — (ZIP) [file pone.0230905.s002.zip › S2_File/CD160.tif]

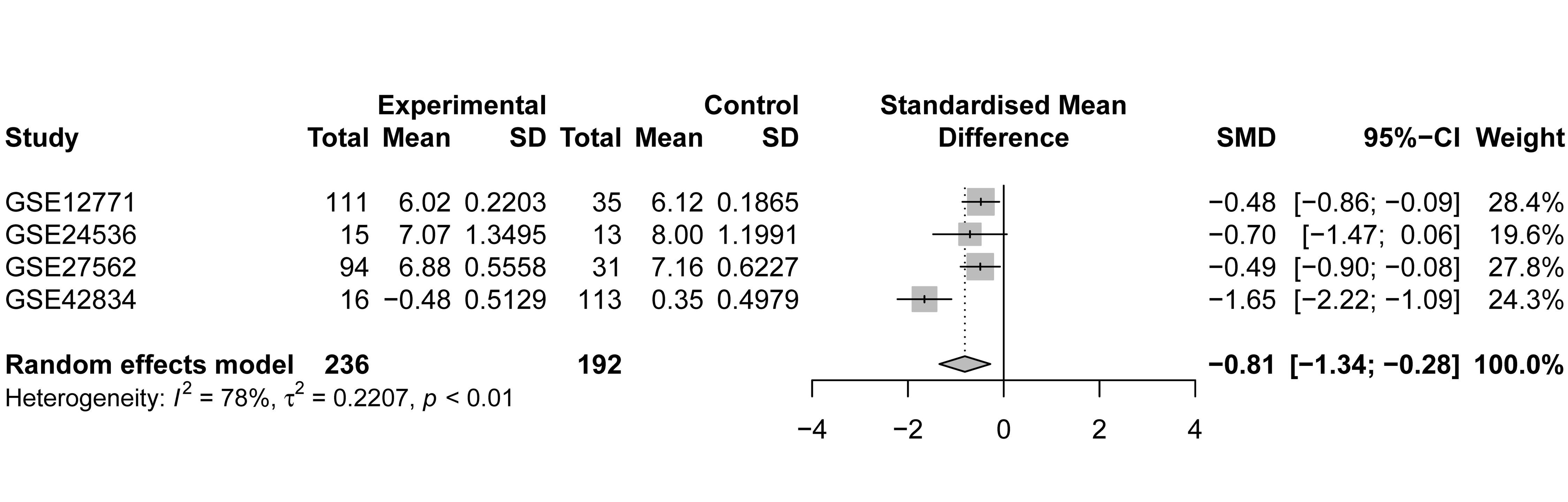

Supplement: S2 Files — (ZIP) [file pone.0230905.s002.zip › S2_File/CD28.tif]

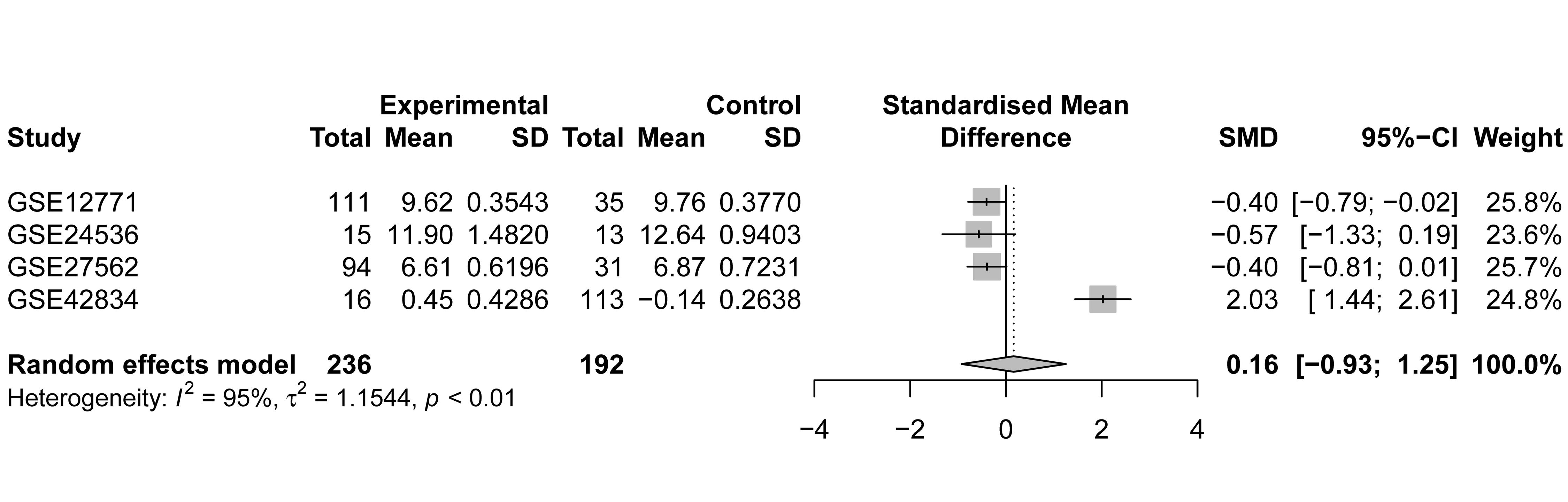

Supplement: S2 Files — (ZIP) [file pone.0230905.s002.zip › S2_File/CD53.tif]

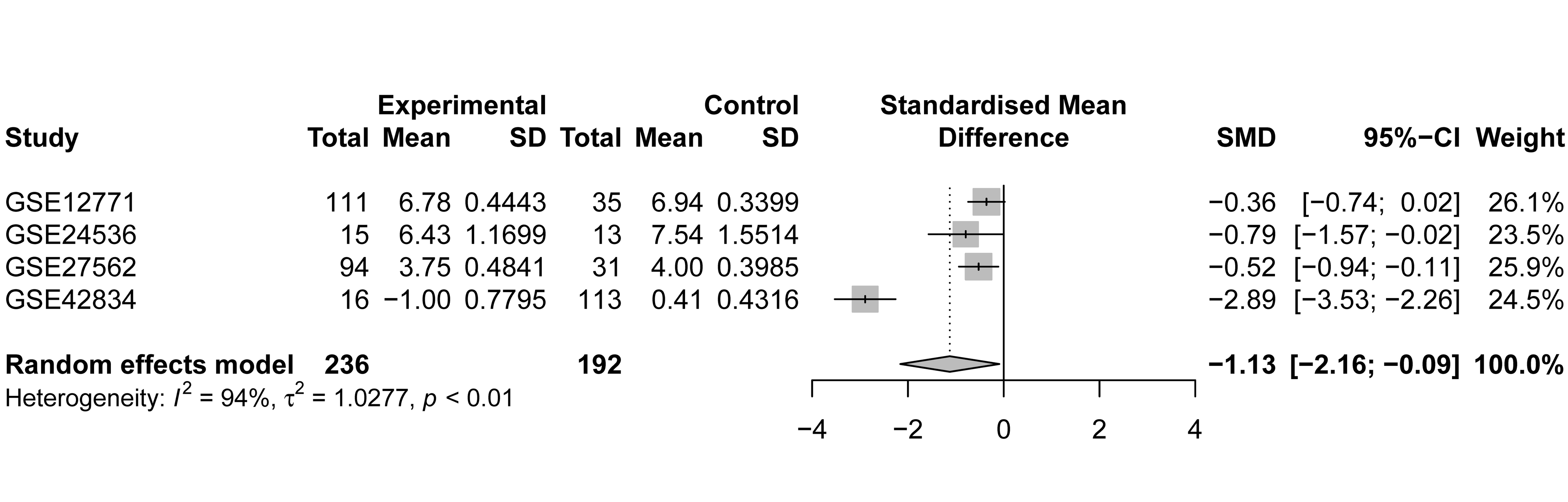

Supplement: S2 Files — (ZIP) [file pone.0230905.s002.zip › S2_File/NMT2.tif]

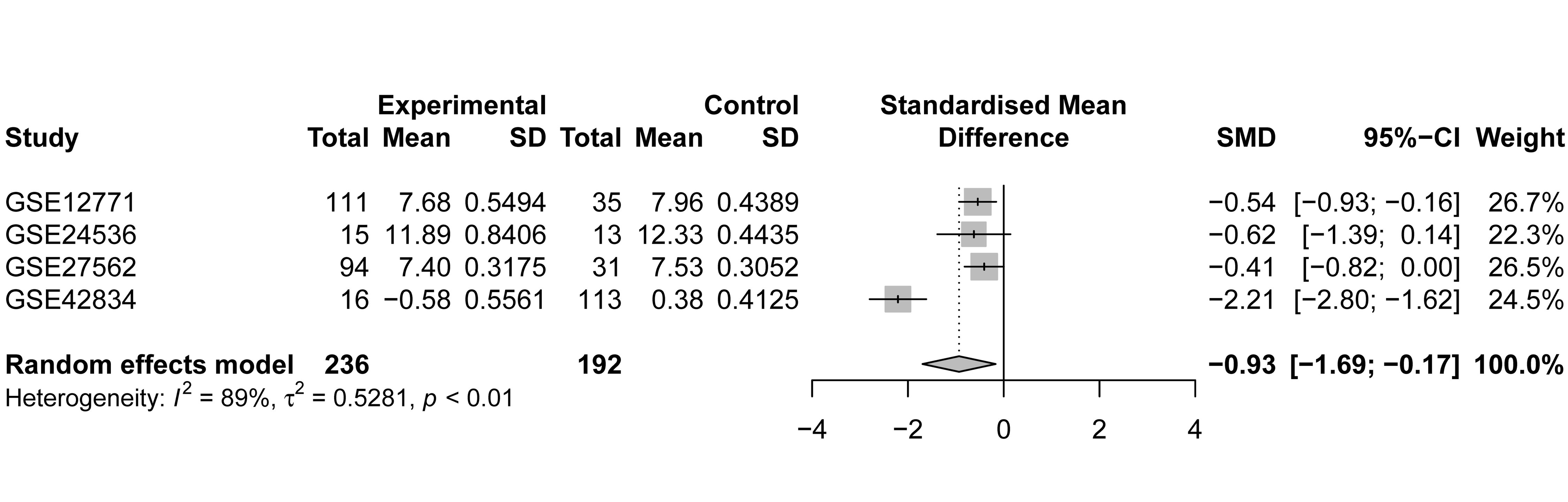

Supplement: S2 Files — (ZIP) [file pone.0230905.s002.zip › S2_File/PLCG1.tif]

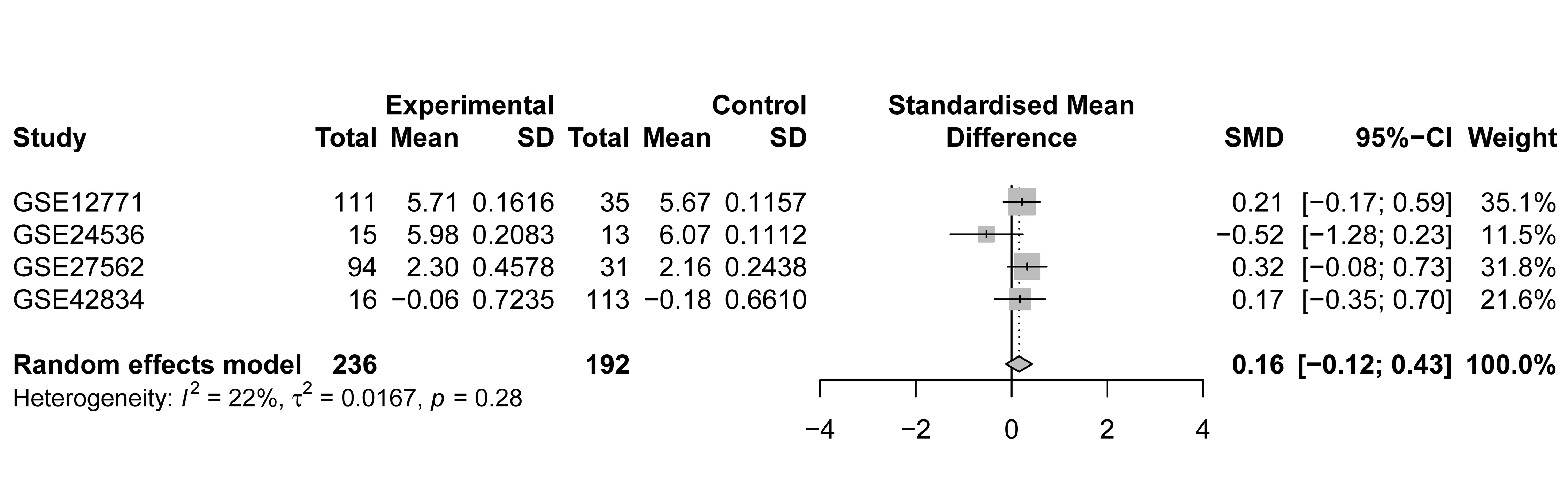

Supplement: S2 Files — (ZIP) [file pone.0230905.s002.zip › S2_File/PLSCR4.tif]

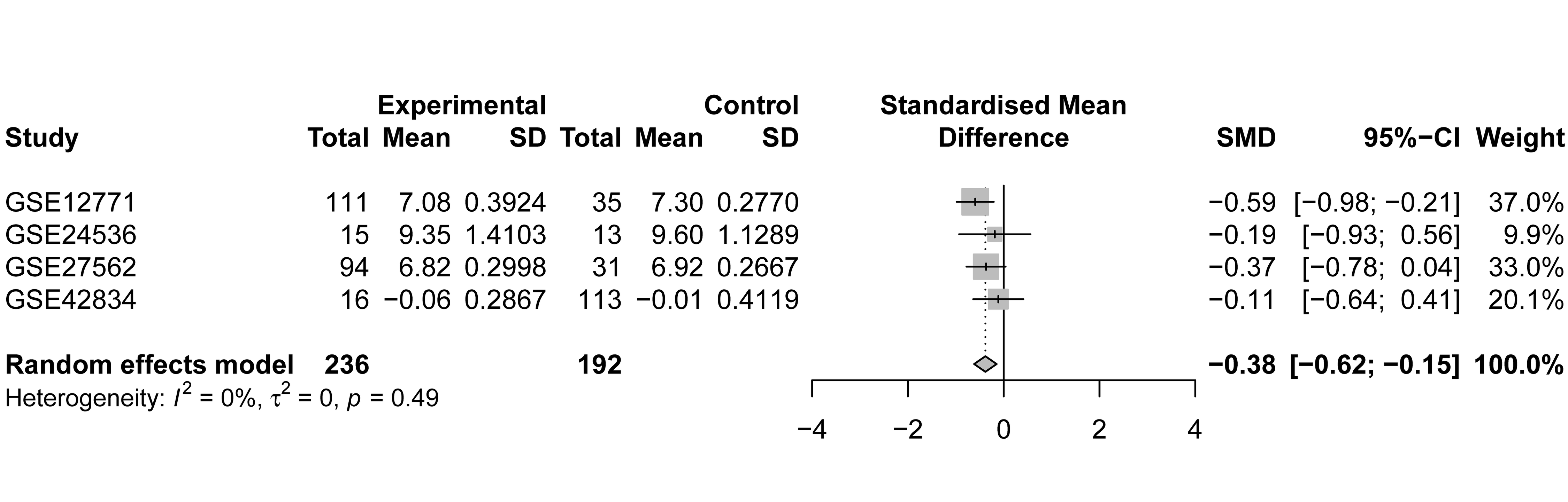

Supplement: S2 Files — (ZIP) [file pone.0230905.s002.zip › S2_File/PMVK.tif]

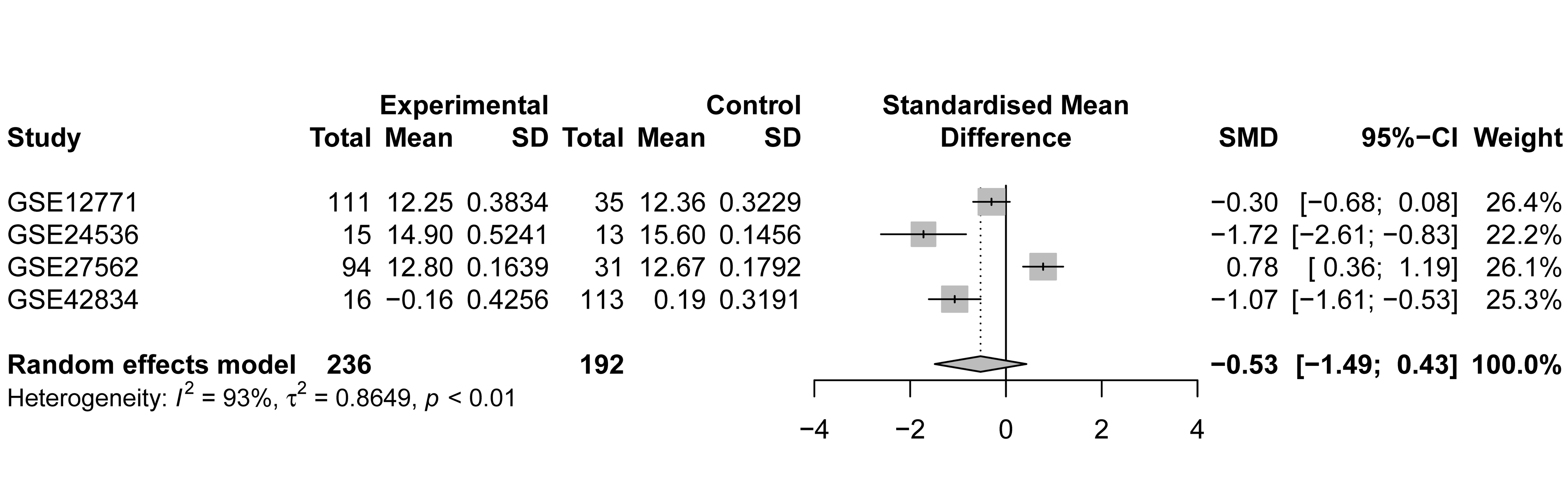

Supplement: S2 Files — (ZIP) [file pone.0230905.s002.zip › S2_File/RPL12.tif]

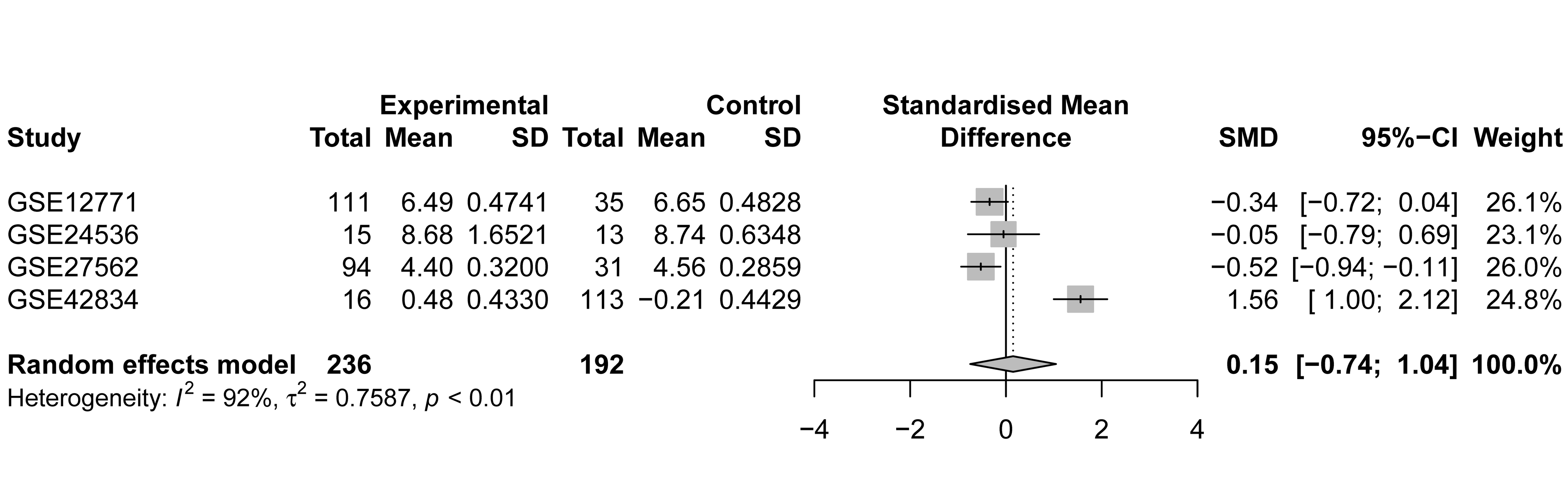

Supplement: S2 Files — (ZIP) [file pone.0230905.s002.zip › S2_File/TNFSF14.tif]
