# Supplementary material for: Identification of human peripheral blood monocyte gene markers for early screening of solid tumors
Source: PLoS One. 2020 Mar 30;15(3):e0230905. doi: 10.1371/journal.pone.0230905 (PMC7105127; doi:10.1371/journal.pone.0230905)
Supplement: S2 Table — (DOCX) [file pone.0230905.s004.docx]

| Additional Table 2. 285 selected genes from reported genes and common genes | | | | |
| --- | --- | --- | --- | --- |
| gene.name | Zscore | Pvalue | pval_teststatistic | es_teststatistic |
| SLC1A5 | 1.231134447 | 0.218272584 | 2.333815113 | -2.325698418 |
| CHD2 | -0.024646855 | 0.980336646 | 2.254873996 | -2.195627684 |
| INPP5D | -1.868272379 | 0.061724115 | 2.121493248 | -2.153578457 |
| IFI44 | -1.677897967 | 0.093367019 | 2.014496134 | -2.053317829 |
| ANXA1 | -0.17866908 | 0.858197549 | 1.787553714 | -1.922907752 |
| OAS1 | -16.88020092 | 6.29E-64 | 2.277089104 | -1.836146138 |
| PLSCR1 | 3.698260749 | 0.000217082 | 1.597957795 | -1.741262816 |
| IFI44L | -0.096112429 | 0.92343128 | 1.84501702 | -1.694007951 |
| CCR2 | -3.544533863 | 0.000393308 | 1.698839661 | -1.631009414 |
| NFIL3 | 15.23344338 | 0 | 1.96482823 | -1.612938811 |
| SLC16A3 | 1.637070736 | 0.10161569 | 1.576742953 | -1.593645525 |
| GBP5 | 2.631421635 | 0.008502847 | 1.387609575 | -1.546709932 |
| LRG1 | 0.883851477 | 0.376776394 | 1.828426233 | -1.535941785 |
| NMI | -0.99676277 | 0.318879674 | 1.639815561 | -1.484809786 |
| LAG3 | 2.473131576 | 0.013393482 | 1.43731786 | -1.463313146 |
| KLRG1 | 4.406461067 | 1.05E-05 | 2.111287754 | -1.461892304 |
| RPL4 | 1.098397653 | 0.272030887 | 1.716014271 | -1.426405591 |
| ARHGDIB | -0.337166386 | 0.735991475 | 1.655833662 | -1.325017352 |
| IFITM3 | -0.157918405 | 0.874521095 | 1.244312848 | -1.307502039 |
| KLRD1 | 1.449963052 | 0.147068823 | 1.338168202 | -1.27045679 |
| PIK3CD | 0.181301891 | 0.856130622 | 1.755943098 | -1.257231548 |
| TAP2 | 0.023147474 | 0.981532637 | 1.418284532 | -1.226093695 |
| CD86 | -11.67298977 | 1.75E-31 | 1.380196808 | -1.209003192 |
| KLRC2 | 4.311917367 | 1.62E-05 | 1.158303899 | -1.139251362 |
| BRCA1 | 0.386743208 | 0.698946325 | 1.13650721 | -1.116667183 |
| FDPS | 0.463211695 | 0.643212634 | 1.005985898 | -1.11494806 |
| TRIM23 | 1.19892727 | 0.230556227 | 1.062184223 | -1.094209241 |
| OSBPL10 | 0.04711217 | 0.962423828 | 1.189619216 | -1.093298257 |
| TMEM41A | -1.87302084 | 0.061065507 | 1.636161275 | -1.082598688 |
| COBLL1 | -0.041714928 | 0.966725953 | 1.733542787 | -1.051649208 |
| TLR4 | 1.029927977 | 0.303043816 | 1.217903502 | -1.045121758 |
| C9orf66 | -1.250278118 | 0.211197969 | 0.96060007 | -1.017844972 |
| RPL7 | NA | NA | 0.89780167 | -1.011341569 |
| ACTR2 | 36.96925923 | 0 | 1.42071112 | -1.002918397 |
| SLA | 0.669757925 | 0.503012119 | 2.714506275 | -0.993820868 |
| TLR7 | -0.219527686 | 0.826239015 | 1.031722104 | -0.988462021 |
| HERC5 | -1.745700844 | 0.080862948 | 0.978428971 | -0.983847365 |
| BRE | -0.186844946 | 0.851782206 | 1.055494259 | -0.957284582 |
| CCL2 | 0.223798179 | 0.822914347 | 1.049474485 | -0.945940066 |
| PCNA | -1.261522603 | 0.207120622 | 1.445107148 | -0.901637383 |
| USP9Y | 1.866326597 | 0.061995686 | 0.891908628 | -0.882458964 |
| USP48 | -1.140938282 | 0.253895607 | 0.928467018 | -0.862719956 |
| CCR1 | -1.914714037 | 0.055528982 | 1.541149069 | -0.860965301 |
| RPS25 | 0.567303016 | 0.570508328 | 0.463748155 | -0.80498431 |
| GBP1 | -1.79011833 | 0.073434891 | 1.410591708 | -0.78897099 |
| RALBP1 | -0.09194281 | 0.926743478 | 2.161007346 | -0.788744824 |
| YY1 | 3.479284537 | 0.000502754 | 2.359486034 | -0.782152331 |
| ATP5E | 0.530626383 | 0.595677709 | 1.541241028 | -0.78063464 |
| LGALS1 | -2.238770383 | 0.02517086 | 0.802715572 | -0.757991942 |
| SNX16 | -1.24134425 | 0.214478605 | 0.591603163 | -0.747767967 |
| ISG15 | -1.575409482 | 0.115161957 | 0.535926494 | -0.687713663 |
| CXCL8 | -0.416692517 | 0.676903329 | 0.720068399 | -0.678141811 |
| OAS3 | -0.851368541 | 0.394564661 | 0.683314686 | -0.661040351 |
| ITGB8 | -0.349282739 | 0.726877056 | 0.936001122 | -0.657871033 |
| BLNK | -0.27996435 | 0.779504856 | 0.047169312 | -0.649520677 |
| ATXN1 | -2.206344396 | 0.027359895 | 0.645094509 | -0.639763944 |
| TGFB2 | 0.840908727 | 0.400399069 | 0.017203327 | -0.61279486 |
| BAIAP2 | NA | NA | 0.09640387 | -0.588916812 |
| PSME2 | -2.236249728 | 0.025335417 | 0.594262833 | -0.586587175 |
| DYNC1LI2 | -2.259086167 | 0.023878025 | 0.783253177 | -0.537988758 |
| TXN | -1.304250244 | 0.192148274 | 0.661891294 | -0.527850163 |
| ITM2B | -1.237623056 | 0.215855862 | 0.936738743 | -0.522730818 |
| GZMB | -0.632637572 | 0.526970346 | 0.491393774 | -0.519421023 |
| PPBP | 2.68198472 | 0.00731868 | 0.727660759 | -0.518166094 |
| C8B | 1.784485039 | 0.074344884 | 0.482490129 | -0.515950489 |
| CCR4 | 1.593978329 | 0.110940889 | 1.402345058 | -0.507857929 |
| RGS1 | -0.093281025 | 0.925680307 | 0.779671093 | -0.502197699 |
| AKAP13 | -0.640257831 | 0.522004991 | 0.407195831 | -0.483473955 |
| EGF | 0.800545963 | 0.423394544 | 0.508427918 | -0.482306076 |
| PTGER2 | -2.510800338 | 0.012045781 | 1.316258044 | -0.460556249 |
| IFIT3 | -2.01352203 | 0.04405974 | 1.024282179 | -0.431930007 |
| STAT1 | 2.721650981 | 0.006495671 | 1.177088511 | -0.408562341 |
| CXCR3 | -1.182902191 | 0.236847913 | 0.491454415 | -0.386979741 |
| EHD2 | 0.655151738 | 0.512370064 | 0.473830838 | -0.386029739 |
| C19orf12 | -0.035675645 | 0.97154099 | 0.278234594 | -0.379518472 |
| TNFRSF1B | -1.577742164 | 0.114624855 | 0.462798728 | -0.378266219 |
| CCL16 | 2.754037189 | 0.005886508 | 0.455881747 | -0.373467508 |
| CXCR2 | -2.395362983 | 0.016603919 | 0.358424421 | -0.354130364 |
| IRF2 | -3.326578052 | 0.000879194 | 0.422286982 | -0.311741195 |
| NFE2L2 | 0.234509556 | 0.814589415 | 1.111493861 | -0.308686722 |
| RGS2 | -0.071578403 | 0.942937428 | 0.932695256 | -0.30269756 |
| GMFG | -0.06007698 | 0.952094324 | 0.371470014 | -0.288805628 |
| DOK2 | -17.63838034 | 1.25E-69 | -0.594952283 | -0.281671687 |
| RAB18 | -0.969381186 | 0.332355036 | 0.305878548 | -0.27630099 |
| CCL22 | 0.545411881 | 0.585470271 | 0.201677834 | -0.274655045 |
| TIRAP | 1.100291611 | 0.271205086 | 0.285944739 | -0.269874398 |
| IL2RG | -1.064370511 | 0.287160887 | 0.28226066 | -0.246854108 |
| HEXA | -1.055287695 | 0.291293767 | 0.527172472 | -0.229386758 |
| RPL36A | -0.966238808 | 0.333924698 | -0.873504974 | -0.224825634 |
| FBXL14 | -0.782301665 | 0.434037309 | 0.240728038 | -0.223932965 |
| IFNG | 0.791343398 | 0.42874363 | 0.363775339 | -0.215991462 |
| XCL1 | -2.374308082 | 0.017581874 | 0.233239466 | -0.174696443 |
| DDB2 | -1.620504543 | 0.105123939 | 0.075308652 | -0.10047642 |
| RPS24 | NA | NA | 0.344973703 | -0.099578307 |
| RPL14 | 0.300184618 | 0.764036337 | 0.441832201 | -0.093167991 |
| RPL37A | -2.306588257 | 0.021077785 | 1.236577377 | -0.072045003 |
| PTPN6 | -1.477019007 | 0.139670542 | 0.065872916 | -0.067989426 |
| TIA1 | -0.327919346 | 0.742972646 | 0.200788189 | -0.053665385 |
| MAPK14 | -0.935947492 | 0.349300223 | 1.220891838 | -0.047514474 |
| TOP1MT | -0.020858854 | 0.983358249 | 0.932772371 | -0.039415892 |
| DGKZ | -0.430689295 | 0.666694304 | 0.079649493 | -0.033106897 |
| XCL2 | -2.071555463 | 0.038306917 | 0.553422103 | -0.031129557 |
| CRTAM | -2.676227844 | 0.007445601 | 0.314667285 | 0.000714885 |
| SRGAP3 | -0.202732829 | 0.839343862 | 0.111603258 | 0.000754923 |
| PFKFB3 | 1.499157025 | 0.133832901 | 0.563885377 | 0.008630455 |
| CMTM5 | 1.978259519 | 0.047899437 | 0.004342409 | 0.020948979 |
| CASP5 | -3.954401437 | 7.67E-05 | 0.0424478 | 0.051771956 |
| PSPC1 | 0.648283314 | 0.516801724 | 0.246606881 | 0.064469005 |
| PRNP | 0.462426753 | 0.643775318 | 0.214228867 | 0.064884198 |
| CXCL10 | 1.099948174 | 0.271354703 | -0.096859965 | 0.077977009 |
| COPB2 | 0.58670841 | 0.557399559 | 0.098473114 | 0.103789751 |
| PSMD12 | -1.079428211 | 0.280396881 | -0.030320402 | 0.123841526 |
| IFI27 | -1.907212469 | 0.056493079 | -0.440135776 | 0.150328034 |
| RPS29 | -1.409742577 | 0.158615708 | 0.202642957 | 0.153571759 |
| SYK | -0.405103177 | 0.685401672 | 0.278828951 | 0.158201682 |
| CCL5 | 0.388028345 | 0.697995058 | 1.151182035 | 0.167447978 |
| ACAT2 | -1.21707799 | 0.223574544 | 0.335553353 | 0.170873152 |
| CD19 | 0.022428336 | 0.982106277 | -0.015466022 | 0.175310099 |
| HLA-DPA1 | -1.027488114 | 0.304190679 | -0.219278741 | 0.182817646 |
| S100A12 | 2.534184402 | 0.01127094 | 0.330440448 | 0.186937265 |
| FPR1 | -1.04060862 | 0.298057229 | 0.364946831 | 0.193485982 |
| ATP6V0B | -2.089593986 | 0.036654287 | 0.220412779 | 0.204677106 |
| CCDC53 | -1.928850874 | 0.053749378 | -0.090992999 | 0.208761883 |
| CD80 | 0.284282048 | 0.776194242 | -0.310823179 | 0.213422723 |
| KIT | -1.915130039 | 0.055475922 | -0.199729829 | 0.219154444 |
| GNLY | -3.565175299 | 0.000363613 | -0.314559487 | 0.219665913 |
| SYN2 | -1.363703877 | 0.172660783 | -0.437975543 | 0.220777316 |
| RPL17 | 1.095540228 | 0.273280032 | 0.253150293 | 0.230175828 |
| MYADM | -1.487476102 | 0.13688911 | 0.394384909 | 0.232436546 |
| RSAD2 | -4.413794312 | 1.02E-05 | -0.038553672 | 0.235078566 |
| JAK3 | -1.599234668 | 0.10976847 | 0.094621287 | 0.239205688 |
| DNAJB14 | -1.305464151 | 0.191734847 | 0.170830105 | 0.273222601 |
| MX2 | -2.135062394 | 0.032755914 | -0.19676412 | 0.282726619 |
| KRAS | -1.434275362 | 0.151493698 | -0.080032608 | 0.298542169 |
| RNPEPL1 | 0.376471611 | 0.706566317 | -0.005098788 | 0.302502962 |
| MNDA | -2.097530446 | 0.035946645 | 0.874895282 | 0.311124617 |
| ATP5L | -1.301461343 | 0.193100588 | -0.105627106 | 0.313997649 |
| STAT3 | 0.812535 | 0.416484712 | 0.013002935 | 0.315302315 |
| M6PR | -2.228228492 | 0.025865282 | -0.272966624 | 0.31566533 |
| UBA52 | NA | NA | -0.130127568 | 0.319086537 |
| TRAF2 | 0.190799501 | 0.84868268 | -0.420599627 | 0.327014555 |
| RPS4Y1 | 0.978983155 | 0.327588303 | -0.887041191 | 0.330243056 |
| CX3CR1 | -1.692509329 | 0.090548903 | -0.437829917 | 0.342091806 |
| SMAD3 | -0.75950885 | 0.447548222 | -0.491639259 | 0.346631143 |
| PSMC2 | -1.58096729 | 0.113885517 | 0.362601905 | 0.35270373 |
| PNPLA1 | -0.687930505 | 0.491496546 | 0.11463718 | 0.372452654 |
| FOXP3 | -0.465275664 | 0.64173406 | -0.405006596 | 0.374073638 |
| OSBPL7 | 0.931483892 | 0.351603313 | -0.42534361 | 0.38090339 |
| CTSD | -1.599011517 | 0.109818044 | -0.247084943 | 0.386597556 |
| BTG3 | -3.966178143 | 7.30E-05 | -0.247201779 | 0.388998889 |
| SLC22A4 | -2.960823815 | 0.003068174 | -0.061231765 | 0.391689778 |
| IDI1 | -1.065819904 | 0.286505062 | -0.066539719 | 0.393017681 |
| ROGDI | -0.428179185 | 0.66852067 | -0.073586926 | 0.394475675 |
| CXCL13 | -0.050463718 | 0.959752862 | -1.12579521 | 0.410948172 |
| CD2 | -0.572742684 | 0.566818932 | -0.212013886 | 0.420417131 |
| ELK1 | -0.612077056 | 0.540486777 | -0.219773192 | 0.430589451 |
| RPL6 | -0.010025944 | 0.992000588 | -0.036069339 | 0.437066812 |
| PSMC6 | -1.93708248 | 0.052735262 | -0.158853853 | 0.447260529 |
| SLC2A3 | 0.072969248 | 0.941830589 | 0.015671436 | 0.45262272 |
| SNRPE | -1.234721771 | 0.21693407 | -0.294487808 | 0.462387783 |
| EGR2 | -1.66189835 | 0.096533158 | -0.516672098 | 0.467410063 |
| CCR5 | -5.766473427 | 8.09E-09 | -0.631801379 | 0.478185252 |
| SULT1A4 | -1.415758927 | 0.156846099 | -0.416423361 | 0.480083137 |
| ETF1 | -0.874676355 | 0.381750029 | 0.130698167 | 0.489016523 |
| CD163 | -2.107733913 | 0.035054012 | -0.395645691 | 0.503377507 |
| C9orf85 | 0.417246711 | 0.676497962 | -0.542512127 | 0.504852449 |
| CD24 | -2.573869995 | 0.010056805 | -0.535805213 | 0.506670289 |
| RSF1 | -1.284427745 | 0.19899233 | -0.23696432 | 0.514107688 |
| CXCL11 | -2.034408972 | 0.041910378 | 0.137474141 | 0.519135005 |
| BTK | -2.158968934 | 0.030852577 | -0.377488276 | 0.519270277 |
| PTK2 | 0.14892799 | 0.881610455 | -0.384536092 | 0.527470636 |
| EP400 | -0.745789316 | 0.455794695 | -0.646508537 | 0.538175265 |
| BCL2L1 | -1.165976766 | 0.24362384 | -0.500628672 | 0.542100571 |
| PF4 | 1.049130411 | 0.294118101 | -0.85909137 | 0.542375656 |
| CLU | 0.0473805 | 0.96220997 | -0.420439887 | 0.545133411 |
| BAD | -1.240680087 | 0.214723953 | -0.386571614 | 0.551062404 |
| CSTA | -1.918432065 | 0.055056249 | -0.065926699 | 0.552030417 |
| PTP4A2 | -0.933755897 | 0.350429824 | -0.460167723 | 0.566651105 |
| NR4A2 | 0.907716182 | 0.364028192 | -0.321400552 | 0.569357879 |
| IFIT1 | -4.147054353 | 3.37E-05 | -0.368065673 | 0.575027906 |
| ZNF675 | -1.282522799 | 0.199659312 | -0.490417333 | 0.582021812 |
| WDFY1 | -1.255229312 | 0.209395528 | -0.699292103 | 0.582674286 |
| MX1 | -2.060217764 | 0.039377727 | -0.652468028 | 0.587311471 |
| TNFRSF1A | -1.644917975 | 0.099986728 | 0.681727039 | 0.590542851 |
| ITCH | -0.187745811 | 0.851075917 | -0.651462033 | 0.594170622 |
| GRN | -1.831026011 | 0.067096661 | -0.23702287 | 0.605264639 |
| RPS4X | NA | NA | -0.593423342 | 0.615235327 |
| TRIO | 0.129232961 | 0.897173316 | -0.252267416 | 0.615635879 |
| TLR9 | 1.072491781 | 0.283499204 | -0.567534938 | 0.618933255 |
| PTPRN | -1.410129748 | 0.158501375 | -0.528550728 | 0.624891064 |
| RPL11 | NA | NA | -0.496664062 | 0.630307671 |
| CXCL9 | -0.463815798 | 0.642779723 | -0.500408716 | 0.630484754 |
| SLC2A14 | 0.55555391 | 0.578515846 | -0.348925723 | 0.63987107 |
| IFIT2 | -4.052796574 | 5.06E-05 | 0.028183943 | 0.643134475 |
| CD1D | -2.325889652 | 0.020024438 | -0.524574692 | 0.659305223 |
| CBLB | -0.662729443 | 0.507503849 | -0.341863796 | 0.661812365 |
| PRODH | -0.622805568 | 0.533412299 | -0.320827614 | 0.680936463 |
| FKBP5 | -1.201629639 | 0.229507053 | -0.596766992 | 0.688225151 |
| CXCR4 | -1.245236297 | 0.213044904 | -1.243685063 | 0.693293348 |
| IGFBP7 | -3.196378156 | 0.001391646 | -0.216535478 | 0.694441102 |
| C1QC | -0.371064673 | 0.710589363 | -0.445386644 | 0.697145471 |
| CREBBP | -0.130663322 | 0.896041648 | -0.849850542 | 0.720297286 |
| ACAA1 | -1.8971866 | 0.057803315 | -0.756111977 | 0.726309066 |
| MRE11A | -2.540876093 | 0.01105751 | -0.72809823 | 0.740117403 |
| ASCC3 | -3.081586557 | 0.002059006 | -0.673045888 | 0.75717106 |
| DDX3Y | -2.952309897 | 0.003154062 | -0.670281495 | 0.766692243 |
| GUK1 | -1.037754543 | 0.299384345 | -0.731333655 | 0.786248091 |
| ITPKB | -2.256629848 | 0.024031214 | -0.713759853 | 0.800724995 |
| CAMP | -1.278230566 | 0.201168142 | -0.855200336 | 0.801904344 |
| KLRF1 | -0.102417507 | 0.918425288 | -0.581498711 | 0.806520425 |
| HEXIM2 | -0.371246801 | 0.710453717 | -0.78300611 | 0.806762707 |
| TNFRSF9 | -1.598497949 | 0.109932201 | -0.972345764 | 0.825624548 |
| PEMT | -1.573132997 | 0.115688026 | -0.502679819 | 0.847807331 |
| FAM110A | -1.506074742 | 0.132047988 | -0.765415389 | 0.859004598 |
| FES | -0.995825602 | 0.319334889 | -0.846229183 | 0.86753484 |
| CDA | -2.546037588 | 0.010895346 | -0.485500824 | 0.872358021 |
| CYP2J2 | 1.558802996 | 0.119043015 | -0.849676355 | 0.87663501 |
| MTHFS | -1.669377591 | 0.095042572 | -1.438418303 | 0.878563991 |
| CDKN2D | -0.291097996 | 0.770976373 | -1.101607998 | 0.882195342 |
| RPL34 | NA | NA | -0.953613656 | 0.886654075 |
| EIF2AK2 | -2.032798036 | 0.042072932 | -1.095511072 | 0.903593543 |
| SERPINI2 | -0.6462048 | 0.518146731 | -0.847537277 | 0.912606639 |
| DDX17 | -2.610074315 | 0.009052256 | -1.011038768 | 0.914697154 |
| TOMM7 | -0.888350175 | 0.374352418 | -1.090839972 | 0.921646543 |
| NACA | -1.875863461 | 0.060674031 | -1.110578772 | 0.928530547 |
| KCNMA1 | 0.792546329 | 0.428042189 | -0.639440807 | 0.937205146 |
| ACADM | -9.780057939 | 1.37E-22 | -1.143203268 | 0.982750531 |
| LIF | 1.33287507 | 0.182572805 | -0.890880636 | 0.994576393 |
| PI3 | 0.331186596 | 0.740503545 | -1.244941178 | 1.051332197 |
| REST | -0.274746317 | 0.783511144 | -0.923582335 | 1.0668349 |
| SOCS3 | 0.719692137 | 0.471714569 | -1.083529483 | 1.076822935 |
| LEF1 | -3.658238276 | 0.000253955 | -1.214828172 | 1.077869962 |
| CMAS | -4.690461235 | 2.73E-06 | -1.192503278 | 1.079984469 |
| DYRK2 | -2.441332704 | 0.014633167 | -1.212898577 | 1.085322627 |
| RPL26L1 | -2.308833089 | 0.020952845 | -1.207254046 | 1.108539223 |
| RPL15 | 0.094181148 | 0.92496526 | -1.160340607 | 1.108597021 |
| SHC1 | -0.667412614 | 0.504508611 | -1.363022215 | 1.125453514 |
| CCL17 | -0.537058794 | 0.591227003 | -0.993513143 | 1.146487579 |
| BATF | -0.747732537 | 0.454621502 | -1.790458311 | 1.162668835 |
| CCL18 | -0.465287178 | 0.641725815 | -1.045464102 | 1.167805784 |
| CD69 | -2.062227985 | 0.039186036 | -1.214711754 | 1.169109376 |
| PLAA | -2.470377454 | 0.013497055 | -1.340202299 | 1.192808419 |
| LCN2 | -3.447199673 | 0.00056643 | -1.281431672 | 1.207858799 |
| INSIG1 | -2.150503521 | 0.031515408 | -1.651441604 | 1.217922844 |
| SELL | -0.116369372 | 0.907359808 | -1.584218789 | 1.242352917 |
| ZSCAN2 | -1.549781818 | 0.121193893 | -1.456019647 | 1.287173576 |
| KLHDC1 | -1.931557172 | 0.053414186 | -1.356867542 | 1.293069849 |
| RASA1 | -1.186171286 | 0.235554663 | -1.38483795 | 1.300520263 |
| CD72 | -4.522863022 | 6.10E-06 | -1.423061171 | 1.312342196 |
| BANK1 | -3.770371188 | 0.000163005 | -1.222935063 | 1.313651807 |
| PSMC4 | -2.919054527 | 0.003510948 | -1.752102639 | 1.33934909 |
| CD4 | -0.356516311 | 0.721453939 | -1.855959009 | 1.372171382 |
| HDC | 0.971276589 | 0.331410564 | -1.702430936 | 1.409926859 |
| INHBE | -0.120494297 | 0.904091597 | -1.412043967 | 1.426053681 |
| MS4A1 | -1.020237997 | 0.307615601 | -1.641545902 | 1.430419588 |
| IL4R | 0.144949706 | 0.884750581 | -1.380852486 | 1.471709993 |
| SULT1A2 | -0.744919628 | 0.456320309 | -1.508735478 | 1.489579924 |
| CD22 | -2.245297795 | 0.024749022 | -1.47618081 | 1.553607929 |
| CD40 | -3.602629966 | 0.000315014 | -2.474127687 | 1.571749608 |
| CREB1 | -2.72291195 | 0.00647093 | -1.562114438 | 1.571822766 |
| CCR7 | 0.517683264 | 0.604679277 | -1.631159793 | 1.641539537 |
| CD83 | -4.864663058 | 1.15E-06 | -1.898123463 | 1.654571965 |
| NME2 | -0.212489011 | 0.83172555 | -1.620414742 | 1.683809308 |
| GTPBP10 | 2.203128111 | 0.02758572 | -2.341306925 | 1.688466973 |
| BDNF | -1.777562587 | 0.075475723 | -2.051747876 | 1.695282259 |
| PRKCZ | 0.904340127 | 0.365815075 | -2.301047388 | 1.721049346 |
| HIST1H4H | -2.709297383 | 0.006742587 | -2.178995331 | 1.737903683 |
| CTLA4 | -1.299542478 | 0.193757825 | -1.918780021 | 1.743150144 |
| BPGM | -5.176807293 | 2.26E-07 | -1.834792474 | 1.769513773 |
| PLCB3 | -1.702986578 | 0.08857058 | -2.979208687 | 1.775741442 |
| MBTPS1 | -1.948461017 | 0.051359825 | -2.066375926 | 1.833626987 |
| SULT1A1 | 2.070850487 | 0.03837277 | -1.820596698 | 1.837849147 |
| CD79A | -4.330289562 | 1.49E-05 | -2.463796399 | 1.846562724 |
| NELL2 | -0.994027717 | 0.320209374 | -2.090912446 | 1.856433523 |
| LTF | 1.224671603 | 0.220698978 | -1.892574209 | 1.885187228 |
| NMT2 | -2.438883603 | 0.014732713 | -2.790448549 | 1.891594063 |
| PMVK | 0.306227477 | 0.759431453 | -3.246965146 | 1.937950945 |
| PLCG1 | 0.529391658 | 0.596533784 | -2.093890116 | 1.944731563 |
| CD160 | -1.490448356 | 0.136106386 | -2.042633225 | 2.09544499 |
| CD53 | -1.908485255 | 0.056328527 | -2.627046434 | 2.120768653 |
| C9orf64 | -1.89105626 | 0.058616834 | -2.980872154 | 2.285266866 |
| CD28 | -2.731109951 | 0.006312141 | -2.463065346 | 2.475447811 |
| TNFSF14 | -3.317208517 | 0.000909217 | -2.874148982 | 2.47651421 |
| RPL12 | 0.728021612 | 0.466600357 | -2.763822056 | 2.851129239 |
| PLSCR4 | -4.223187699 | 2.41E-05 | -3.105852833 | 3.140376984 |
