# Supplementary material for: Identification of human peripheral blood monocyte gene markers for early screening of solid tumors
Source: PLoS One. 2020 Mar 30;15(3):e0230905. doi: 10.1371/journal.pone.0230905 (PMC7105127; doi:10.1371/journal.pone.0230905)
Supplement: S3 Table — (DOCX) [file pone.0230905.s005.docx]

| Additional Table 3. KEGG pathway enrichment analysis of Cluster 2 | | | | | | | |
| --- | --- | --- | --- | --- | --- | --- | --- |
| geneSet | description | C | O | E | R | pValue | FDR |
| hsa05160 | Hepatitis C | 131 | 2 | 0.035078 | 57.01527 | 3.05E-04 | 0.033518 |
| hsa05162 | Measles | 132 | 2 | 0.035346 | 56.58333 | 3.10E-04 | 0.033518 |
| hsa04217 | Necroptosis | 162 | 2 | 0.043379 | 46.10494 | 4.68E-04 | 0.033518 |
| hsa05164 | Influenza A | 171 | 2 | 0.045789 | 43.67836 | 5.21E-04 | 0.033518 |
| hsa05168 | Herpes simplex infection | 185 | 2 | 0.049538 | 40.37297 | 6.10E-04 | 0.033518 |
| hsa05167 | Kaposi sarcoma-associated herpesvirus infection | 186 | 2 | 0.049806 | 40.15591 | 6.17E-04 | 0.033518 |
| hsa05169 | Epstein-Barr virus infection | 201 | 2 | 0.053822 | 37.1592 | 7.21E-04 | 0.033564 |
| hsa05165 | Human papillomavirus infection | 339 | 2 | 0.090775 | 22.03245 | 0.002054 | 0.08371 |
| hsa05321 | Inflammatory bowel disease (IBD) | 65 | 1 | 0.017405 | 57.45385 | 0.017331 | 0.52885 |
| hsa04917 | Prolactin signaling pathway | 70 | 1 | 0.018744 | 53.35 | 0.018658 | 0.52885 |
| hsa05140 | Leishmaniasis | 74 | 1 | 0.019815 | 50.46622 | 0.019718 | 0.52885 |
| hsa05212 | Pancreatic cancer | 75 | 1 | 0.020083 | 49.79333 | 0.019984 | 0.52885 |
| hsa04658 | Th1 and Th2 cell differentiation | 92 | 1 | 0.024635 | 40.59239 | 0.024485 | 0.52885 |
| hsa04933 | AGE-RAGE signaling pathway in diabetic complications | 99 | 1 | 0.02651 | 37.72222 | 0.026336 | 0.52885 |
| hsa04620 | Toll-like receptor signaling pathway | 104 | 1 | 0.027848 | 35.90865 | 0.027656 | 0.52885 |
| hsa04625 | C-type lectin receptor signaling pathway | 104 | 1 | 0.027848 | 35.90865 | 0.027656 | 0.52885 |
| hsa04659 | Th17 cell differentiation | 107 | 1 | 0.028652 | 34.90187 | 0.028448 | 0.52885 |
| hsa05145 | Toxoplasmosis | 113 | 1 | 0.030258 | 33.04867 | 0.030032 | 0.52885 |
| hsa04919 | Thyroid hormone signaling pathway | 116 | 1 | 0.031062 | 32.19397 | 0.030823 | 0.52885 |
| hsa04380 | Osteoclast differentiation | 128 | 1 | 0.034275 | 29.17578 | 0.033984 | 0.553932 |
| hsa05161 | Hepatitis B | 144 | 1 | 0.038559 | 25.93403 | 0.03819 | 0.592857 |
| hsa04630 | JAK-STAT signaling pathway | 162 | 1 | 0.043379 | 23.05247 | 0.042912 | 0.604227 |
| hsa04141 | Protein processing in endoplasmic reticulum | 165 | 1 | 0.044183 | 22.63333 | 0.043697 | 0.604227 |
| hsa04621 | NOD-like receptor signaling pathway | 168 | 1 | 0.044986 | 22.22917 | 0.044483 | 0.604227 |
| hsa05152 | Tuberculosis | 179 | 1 | 0.047931 | 20.86313 | 0.04736 | 0.617577 |
| hsa04062 | Chemokine signaling pathway | 189 | 1 | 0.050609 | 19.75926 | 0.049972 | 0.626574 |
| hsa05203 | Viral carcinogenesis | 201 | 1 | 0.053822 | 18.5796 | 0.053102 | 0.641155 |
