# Supplementary material for: Identification of human peripheral blood monocyte gene markers for early screening of solid tumors
Source: PLoS One. 2020 Mar 30;15(3):e0230905. doi: 10.1371/journal.pone.0230905 (PMC7105127; doi:10.1371/journal.pone.0230905)
Supplement: S4 Table — (DOCX) [file pone.0230905.s006.docx]

| Additional Table 4. KEGG pathway enrichment analysis of Cluster 3 | | | | | | | |
| --- | --- | --- | --- | --- | --- | --- | --- |
| geneSet | description | C | O | E | R | pValue | FDR |
| hsa05164 | Influenza A | 171 | 4 | 0.16026242 | 24.9590643 | 8.80E-06 | 0.00286888 |
| hsa05160 | Hepatitis C | 131 | 3 | 0.12277413 | 24.4351145 | 1.75E-04 | 0.01948532 |
| hsa05162 | Measles | 132 | 3 | 0.12371134 | 24.25 | 1.79E-04 | 0.01948532 |
| hsa04621 | NOD-like receptor signaling pathway | 168 | 3 | 0.1574508 | 19.0535714 | 3.66E-04 | 0.02983707 |
| hsa05168 | Herpes simplex infection | 185 | 3 | 0.17338332 | 17.3027027 | 4.86E-04 | 0.03170619 |
| hsa05169 | Epstein-Barr virus infection | 201 | 3 | 0.18837863 | 15.9253731 | 6.20E-04 | 0.03371054 |
